# Fluctuations in Sequential Many-Alternative Decisions Reveal Strategies Beyond Immediate Reward Maximisation

**DOI:** 10.5334/joc.467

**Published:** 2025-11-18

**Authors:** Alice Vidal, Francesco Damiani, Alireza Valyan, Salvador Soto-Faraco, Rubén Moreno-Bote

**Affiliations:** 1 Center for Brain and Cognition, and Department of Information and Communication Technologies, Universitat Pompeu Fabra, Barcelona, Spain; 2 Department of Experimental and Health Sciences, Universitat Pompeu Fabra, Barcelona, Spain; 3 Allameh Tabataba’i University, Tehran, Iran; 4 Institució Catalana de la Recerca i Estudis Avançats (ICREA), Barcelona, Spain; 5 Serra Húnter Fellow Programme, Universitat Pompeu Fabra, Barcelona, Spain

**Keywords:** Decision making, Mathematical modelling, Motivation, Reward processing, Executive functions

## Abstract

Humans are strategic animals. We constantly make prospective choices, allocating limited resources in situations of uncertain, future outcomes. The management of our finite monthly budget, financial investments, or the allocation of time to the different questions in an exam are just a few examples. In these scenarios, both decision-making and resource allocation tend to fluctuate over time even under invariable set of constraints. However, it is unclear whether these fluctuations affect performance and whether they underlie additional objectives beyond pure reward maximisation. We address these questions using the breadth-depth dilemma, a novel ecological protocol where participants engage in sequential multiple-choice scenarios characterised by limited capacity. We designed two experimental environments. In one environment, optimal performance, formalised with an ideal allocator model, is associated with homogeneous resource allocation across consecutive choices. In contrast, the other environment entails that fluctuating resource allocation leads to greater expected rewards. Our study evaluates participants’ adherence to these scenarios and measures fluctuations as deviation from homogeneous allocations. The results revealed that participants’ behaviour fluctuates more than optimal, but critically, behavioural fluctuations adapt to the available capacity and the environmental context. Moreover, our findings unveil pronounced sequential strategies, such as save-for-later and reward history-dependent choice, further implying that these strategies contribute to decision variability. An extension of the optimal allocator model demonstrates that the characteristic excess fluctuations facilitate better-informed future choices (information gain), reduce uncertainty (risk avoidance), and generate diverse potential strategies (entropy seeking). Although having a modest impact on performance, these strategies may reflect advantageous behaviours in the long run under ever changing real-world environments.

## Introduction

Ongoing behaviour is a highly dynamic and variable process, shaped by a multitude of internal and external factors. For example, the variety in the choice and sequence of words used to convey a single idea is vast. As a result, we can easily detect when someone delivers a rehearsed talk, lacking the natural fluctuations that characterise spontaneous speech. This inherent variability extends beyond linguistic expression; it is a fundamental aspect of human behaviour that cannot be reconciled through numerous repetitions of the same action or controlled experimental settings (Rahnev & Denison, 2018; Renart & Machens, 2014). When faced with the same decision multiple times, we might undergo different internal processes each time, consider information differently and eventually make slightly different choices, even under ostensibly identical circumstances. Such behavioural variability suggests that the mechanisms underlying our decisions may respond to aims beyond mere reward maximisation, perhaps embodying more nuanced, adaptive strategies. Whether behavioural variability can be fully framed within a reward-maximisation framework, or instead arises from broader adaptive strategies, remains an open question. In this work, we address that question by probing the relation between behavioural variability and performance via a novel paradigm based on a recently developed decision-making task (Vidal et al., 2022).

### Capturing complex adaptive behaviour within the Bread-Depth dilemma

One way to formalise such behavioural flexibility is through the breadth-depth (BD) paradigm, which models real-world dilemmas in which cognitive or material resources, such as time, effort, or attention, are limited and must be strategically allocated among multiple alternatives to maximise expected rewards (Moreno-Bote et al., 2020). The BD trade-off applies to various situations where search capacity must be allocated in advance before accessing the collected information. This dilemma formalises a very common problem in many real-world contexts, for example when planning a vacation, investing money, or shopping at a new local market. Consider this last scenario, products cannot (usually) be consumed on-site, and one must purchase fruits and vegetables before trying them out. Individuals face a choice: they can either buy numerous products from a few vendors to obtain a precise estimation of product quality from that one vendor (depth) or opt to purchase a few products from many different vendors (breadth), thereby increasing the likelihood of finding a good-quality vendor but at the cost of maybe not being able to identify the best one. The BD task captures this trade-off: in each trial, participants must decide in advance how to allocate a fixed budget of search capacity across multiple unknown options, with no outcome feedback until the allocation is complete. This distinguishes the BD task from the classical Exploration-Exploitation (EE) dilemma (Berger-Tal et al., 2014), where feedback guides learning sequentially and past outcomes, such as unexpectedly low rewards (R. F. Green, 1984; Stephens & Krebs, 1986), inform future choices about whether exploiting a known alternative or exploring a new one instead. As a result, variability in the EE dilemma is restricted to the transition between exploration and exploitation and, as mentioned, is largely dependent on previous reward received. In contrast, the BD framework allows for an examination of decision variability in a more complex, high-dimensional space, independently of reward history.

### Humans follow close-to-optimal solutions within the BD dilemma

Additionally, the BD paradigm has the advantage of being analytically tractable, allowing normative solutions to be derived under ideal observer assumptions (Moreno-Bote et al., 2020). These optimal policies dictate that when little capacity is available, all possible alternatives should be sampled (breadth). As capacity available increases, resources should be split on a relatively small number of alternatives so that the information gathered is more focused (depth). This trade-off is sensitive to the environment richness. When most of the alternatives are associated with high reward (rich environment), depth should be favoured other breadth, meaning resources should be concentrated on a few alternatives in order to determine which is the best one. Conversely, when more of the alternatives are associated with low reward (poor environment), resources should be split across many alternatives (breadth), increasing the chances of encountering at least one satisfactory option. Empirical work shows that human decision makers can approximate closely this policy structure, adjusting the breadth and depth of their search in relation to search capacity available and environmental richness (Vidal et al., 2022). Thus the BD framework provides a valuable tool for assessing the influence of task parameters on decision strategies, and for studying their departures from the known optimal solutions.

### Behavioural variability and performance

Indeed, despite the existence of an accessible optimal strategy or constant outcome (Sang et al., 2020), humans’ decisions remain highly variable and individuals often fluctuate around optimality. Traditionally, such variability has been attributed to noise in sensory or decision-making systems (Gigante et al., 2009; Drugowitsch et al., 2012; Smith & Ratcliff, 2004; Renart & Machens, 2014). However, the impact of behavioural fluctuations on performance and their underlying objectives remain poorly understood. Emerging work challenges the interpretation of variability as mere noise, suggesting that behavioural fluctuations can reflect adaptive strategies that accommodate contextual uncertainty, changing internal states, or latent goals (Waschke et al., 2021; Dhawale et al., 2017). To assess whether this holds in resource allocation settings like BD, we require experimental designs that allow participants not only to decide *how* to allocate capacity, but also *when* and *how much* to allocate across time. Indeed, granting two additional degrees of freedom in forming decision strategies, provides increased strategic flexibility, potentially revealing more complex decision rules guiding final choices. Given constraints of limited resources and a fixed horizon, participants are forced to plan carefully. By comparing their implemented strategies with known analytic optimal solutions, we can quantify fluctuations and investigate the sources of behavioural variability.

### Extension of the BD dilemma to study behavioural variability

To this end, we developed an extension of the BD dilemma in which participants sequentially allocate a finite budget of search capacity over a series of trials. This modification introduces an additional layer of decision making: instead of operating under fixed per-trial constraints, participants may now vary how much capacity they expend on each decision, adopting minimal or null investment trials or concentrate resources strategically. This flexible structure enables us to assess two forms of variability—within-trial (breadth vs depth) and across-trial (total capacity invested in each trial)—and examine their relationship to task constraints and reward expectations. By allowing endogenous modulation of investment over time, this paradigm offers a novel window into behavioural fluctuations that may serve computationally adaptive purposes.

### Measuring fluctuations in behaviour

We first examine how participants manage finite search resources (capacity) across various alternatives within a choice (BD trade-off) depending on the probability of success of the alternatives (environment richness), with the goal to replicate previous findings (Vidal et al., 2022). Subsequently, we delve into an exploration of how experimental conditions, including the number of consecutive choices (horizon) and the total number of resources accessible in the block, influence this BD trade-off.

Our focus then shifts towards participants’ resource allocation across successive choices: are finite resources distributed uniformly among the choices or exhibit fluctuations? The introduction of voluntary capacity control is essential for distinguishing between randomness and purposeful variation. Fluctuations can then be measured in terms of deviations in the capacity allocated across trials from an uniform allocation (same capacity allocated in all trials). To explore how variability is coupled with task performance, we implement two environmental contexts in which fluctuating is either optimal or, in contrast, should be avoided. By decoupling fluctuations from expected performance, we aim to identify potential underlying cognitive processes driving behavioural variability. We hypothesise that when gathering information, humans are not purely reward maximisers; instead, they sacrifice optimality (in the sense of reward maximisation) to seek valuable information to facilitate future decisions and explore possible existing strategies while considering the level of risk and uncertainty one can afford.

### The optimal model predicts behavioural fluctuations

The optimal model under this extended task architecture predicts that trial-to-trial variability in capacity investment should increase in environments with low expected value (poor environments), conditions under which focusing on a subset of choices where more resources will be allocated is more advantageous in order to find one of the rare alternatives associated with higher rewards. In contrast, in rich environments, where high rewarding alternatives are frequent, the optimal policy predicts a uniform investment of resources across choices. These predictions allow us to ask whether observed fluctuations reflect sensitivity to task structure or instead diverge in a way that reveals hidden costs, constraints, or motives.

### The extended optimal model: explaining the causes of excess fluctuations

We expected, and our results confirmed, that human behaviour under the extended BD task frequently departs from these normative predictions and participants often display greater variability than is strictly optimal.

We introduced an extended computational model incorporating additional motives driving behaviour. We now allow the new optimal strategy to balance different behavioural strategies, together with the classic reward maximisation one. More specifically, in this extended setting, variable behaviour might arise from entropy and/or information seeking policies, as well as intrinsic biases (such as risk aversion), going beyond the interpretation of variability as mere noise. Notably, this is in line with recent evidence (Garrett et al., 2013; Dhawale et al., 2017; Sternad, 2018; Markowitz et al., 2023), suggesting that behavioural variability may serve purposes beyond immediate reward maximisation (von Neumann & Morgenstern, 1944).

The entropy seeking hypothesis is in line with recent computational work in Reinforcement Learning (maximum occupancy principle, Ramírez-Ruiz et al., 2024) and might reflect a core feature of behaviour, shared across humans and animals. For example, random behaviour has been shown to be useful to evade predators (Evans et al., 2019) or to enhance cognitive flexibility (Dajani & Uddin, 2015; Uddin, 2021) and foster creativity – with advantages in numerous contexts (Mooneyham & Schooler, 2013). In our extended task, an entropy seeking agent would tend to allocate different number of resources in each choice, possibly trying to adapt to unpredicted changes in the environment.

On the other side, variability might arise from an information seeking tendency (Gottlieb, 2012), as seen in monkeys prioritising information over reward (Blanchard et al., 2015) and mice engaging in exploration at the expense of perceptual choices (Pisupati et al., 2021). Note that throughout the paper, we use the term “exploration” to refer broadly to the act of seeking information, rather than the specific exploration process defined in the EE dilemma. Recent studies incorporate the idea that humans do not only value alternatives based on their expected associated reward but also on their associated information (Cogliati Dezza et al., 2017). In practice humans have indeed been found to orient their choice towards uncertain alternatives (Schulz et al., 2019; Alméras et al., 2021) or alternatives associated with information believed to facilitate future actions (Kelly & Sharot, 2021; Sharot & Sunstein, 2020). In the extended BD dilemma, a decision-maker pursuing informative sampling would try to increase the chance that one alternative stands out as evidently better than the rest, simplifying its eventual selection.

Thirdly, human may adapt their search strategy according to intrinsic needs such as their aversion to risk (Tulloch et al., 2015). For example, in the decision paradigm proposed in this work, a risk adverse participant would avoid leaving a choice to chance by allocating no resources.

What is more, the weight of these motives may be affected by the environmental context (L. Green & Myerson, 2004; Seymour & McClure, 2008). For example, exploring and seeking novel alternatives is enhanced in extended time horizons, affording more opportunities to leverage newly acquired knowledge (Carstensen et al., 1999; Wilson et al., 2014; Averbeck, 2015) which underscores the interconnection between planning and information-seeking (Hunt et al., 2021).

By integrating these latent strategies, the extended model can predict a more diverse range of allocation strategies. For instance a high emphasis on entropy seeking results in stronger deviations from uniform distributions, as participants endeavour to allocate varying numbers of resources across trials. Conversely, high risk aversion might reduce the number of trials where no capacity is allocated (left to chance). However, it does not necessarily diminish fluctuations, as fewer resources (compared to what predicted by a uniform distribution) may still be allocated. Furthermore, sampling strategies may be tailored to facilitate the selection of the final supplier (amongst the sampled ones) by minimising the likelihood of ties where uncertainty is about what action to take is high, although it is associated with lower outcomes. Overall, the extended model repositions variability not as a failure mode but as a potential manifestation of informational and intentional cognitive flexibility.

## Methods

### Participants

Participants were recruited through the CBC lab participants database (https://www.upf.edu/web/cbclab), with the criteria of being fluent in English or Spanish, with age between 18–55 and being proficient with the manipulation of the touch screen as the task was performed on a touch screen laptop. Participants received a monetary compensation which was partly based on their final score at the task (number of good-quality apricots collected), ranging between a minimum of 9.30€ and a maximum of 10.60€ for an hour. Additionally, participants who obtained the three top scores in each environment condition (rich and poor), were rewarded with an additional 20, 10 and 5€, respectively.

Participants were recruited until completing a valid final sample size of 20 participants in each of the two environment conditions (20 males, mean age ± sd: 26.8 ± 8.4 years). This initial sample size was decided in order to detect an effect of the environment on the BD trade-off with a 95% power (Cohen’s d = 1.28, estimated from a previous study – see Vidal et al., 2022) and detect medium to large effect sizes with an 80 % power between and within participants respectively (more details in the preregistration: https://osf.io/4dsma).

Data from an additional 4 participants were discarded before analysis based on the pre-established criterion that they spent less than 90% of the initial capacity (*N_c_*) during the experiment (see bellow). We considered that such behaviour could reflect participants lack of attention or understanding in the task. Another participant was discarded because of a technical problem which prevented him from completing the whole experiment.

### Analyses

Analyses were run using R and MATLAB. Normality of the data was tested using Shapiro tests and homoscedasticity was tested using F tests or Bartlett tests (for more than 2 samples). In cases where it was possible, parametric tests were preferred, otherwise non-parametric tests were used. One sample Wilcoxon tests against the environment averaged outcome (25 and 75 respectively for the poor and rich environment) were used to test whether participant’s final score was significantly higher than chance. For all participants, tests were significant (for *α* = . 05).

## The free Apricot Breadth-Depth task

As the task is similar, methods describing the experimental design were adapted from Vidal and colleagues (2022). This study was pre-registered (https://osf.io/4dsma).

### An experimental design to explore the relation between fluctuations and performance

We developed a variation of the Breadth-Depth Apricot Task (Vidal et al., 2022) to test human search behaviour in sequential multiple economic choice scenarios under limited resources. The task was programmed using MATLAB (R2021b) and run using laptops with touch screens. Participants were initially introduced with a realistic narrative that provided a concrete everyday-life context to aid understanding the task goals and constraints. According to this narrative, at the end of each trial the participant purchases an order of apricots in bulk from one specific supplier, out of many available. The goal is to maximise the amount of good quality apricots accumulated throughout the experiment. Because suppliers vary in the proportion of good quality apricots they serve, participants are given the opportunity to sample suppliers’ goods before the final bulk purchase, by spending some of their coins (capacity) in exchange for sample apricots. There are 20 available suppliers, but the total amount of available coins is limited so sampling all the suppliers is impossible. Based on the sampling outcomes in each trial, participants are to choose the supplier for the final purchase. In this study, we assigned fixed budgets for blocks of several consecutive purchases which can vary in length. Therefore, participants sample a limited number of suppliers at each trial or even choose not to sample any supplier before purchase (skipped trial) in order to save coins for future purchases or because the budget for that block is exhausted, in which case the supplier for the final purchase must be selected randomly.

**Figure 1:**
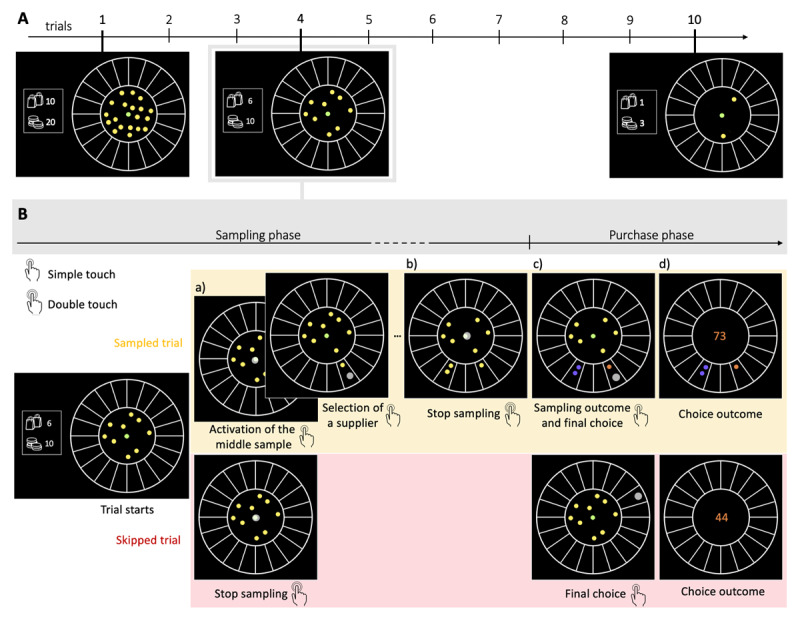
**The BD apricot task with capacity freely allocated amongst trials. A. Example of one block** composed of 10 consecutive choices (trials, *N_trials_* = 10) and with an initial capacity *N_c_* of 20 samples. The capacity ratio *r* of this block represents the initial number of samples divided by the number of trials, in this case *r* = 2. On each trial, the remaining coins are displayed at any time in two ways: written on the left of the wheel (bottom number) and materialised by the yellow and green dots located within the centre of the wheel. For example, at the beginning of the 4^th^ and last trial, the remaining capacity (*N_r_*) is respectively of 10 and 3 samples. The remaining number of purchases (trials) in the block is also constantly displayed on the left of the wheel (top number). **B. Example of one trial**. Participants allocate a limited search capacity (coins) to assess the quality of good apricots in different suppliers (sampling phase). Subsequently, they make a final purchase of 100 apricots from one of the sampled suppliers (purchase phase). Each distinct black section of the wheel represents a different supplier. The initial number of coins per block varies pseudo-randomly from block to block within a finite range (defined by the capacity ratio *r*, multiplied by the number of trials in block *N_trials_*- see Methods). To allocate the coins to suppliers, participants have first to click on the designated active coin displayed at the centre (green dot) and then select the supplier to sample from (panels a) –both touch screen events are indicated by a large grey dot. One of the inactive (yellow) coins is then automatically activated and displayed, in green, at the centre. This sequence repeats until all coins are allocated or until participants end the sampling phase by touching twice the centre coin (panel b). Then, each of the allocated samples turn either orange, representing a good-quality apricot, or purple, representing a bad-quality apricot (panel c). Finally, after this information is revealed, the participant selects one of the sampled suppliers for the final purchase of 100 apricots (with a touch screen, indicated by a large grey dot) and the choice outcome is immediately displayed (panel d). In the case where no coin has been allocated (skipped trial – lower panels), participants select randomly one of the 20 suppliers for the final purchase.

Each trial (purchase) in the task was divided into a sampling phase and a final purchase phase, with participants having the possibility to skip this first phase (skipped trials). The number of coins spent during the sampling phase determines the search capacity of the participant on each trial. We manipulated the block length *N_trials_* (10 or 20 trials) and the average number of coins available per choice in a block of trials (capacity ratio *r = N_c_/N_trials_*: 2, 3 or 4), resulting in an initial capacity within a block *N_c_* from 20 to 80 coins. For instance, in a block with a capacity ratio *r* of 2 and length of 10 trials, a total of 20 coins were initially given (Figure 1A). When characterising the available capacity in a block, we refer to this relative value *r*, independent of the block length, instead of the initial capacity *N_c_*.

A video showing the proceedings of our experimental design is available here. On each trial, the remaining capacity (*N_r_*) is clearly visible with the remaining coins being displayed, at any time, within the centre of the wheel (Figure 1A). In each trial, the coins could be freely allocated one by one to any of the 20 suppliers by clicking the active coin in the middle of the display and then by clicking the desired supplier to sample from (Figure 1B.a). Participants could arbitrarily allocate the coins in a given trial (i.e., all coins to just one supplier, or each coin to a different supplier, or anything in between). Once the desired number of samples has been allocated, participants doubled click on the sample in the centre to end the sampling phase (Figure 1B.b). Only then the samples were revealed (Figure 1B.c) as a binary outcome: either of good (orange) or bad quality (purple) apricots.

On each trial, participants could decide how many coins they would use to gather information about the suppliers (Figure 1B – *sampled trial*), or forgo sampling to save coins and randomly select one supplier (Figure 1B – *skipped trial*) for the final purchase of 100 apricots (Figure 1B.d) at the end of each trial. Participants were encouraged to use all their available coins within the block (any remaining coins were forfeited, as they could not be carried over to the next block). The allocated capacity in the trial n (n = {1, 2, …, *N_trials_*}) is denoted as *C_n_* (*C_n_* = {0, 1, 2,…, *N_r_*}), where *N_r_* is the remaining capacity at that trial \[\left({{N}_{r}}~=~{{N}_{c}}~-~{\sum}_{i=1}^{r-1}{{C}_{i}}\right)\]. We refer generically to *C* as the allocated capacity in a given trial within a block, while *M* stands for the number of sampled alternatives in a given trial.

When sampling, participants used each coin to buy one sample apricot from a chosen supplier, and they could purchase multiple sample apricots from the same supplier to obtain a more accurate estimation of its probability of providing good-quality apricots. At any time, participants had the choice to stop sampling by double clicking on the sample situated at the centre (Figure 1B.b) and then the quality of the sample apricots would be revealed (good: orange or bad: purple; Figure 1B.c). The feedback regarding the sampled alternatives was consequently postponed until after the termination of the sampling, thereby preventing any real-time adjustment or correction of the search strategy. The final bulk purchase had then to be made by choosing from one of the sampled suppliers, therefore completing the trial and proceeding to the next, or to the end of the block.

The sampling outcomes *O_i_* at each supplier *i* (given the range 1 to 20) followed a binomial distribution *O_i_*~*B*(*n_i_*, *p_i_*) where *n_i_* is the number of samples allocated in supplier *i* and *p_i_* is the fraction of good quality apricots in that supplier. While *n_i_* is chosen by the participants, *p_i_* is unknown to them. Based on the information collected, participants could estimate *p_i_*, and based on the estimation could choose amongst the sampled suppliers (and only the sampled ones) to perform a final bulk purchase of 100 apricots from a finally chosen supplier. We prevented participants from selecting a non-sampled supplier to motivate a more careful search as, in case of only negative outcomes, one of the sampled suppliers would have to be selected anyway. The number of good quality apricots contained in the purchase was revealed (Figure 1B.d), and the next trial (purchase cycle) started. The cumulative sum of good-quality apricots collected, as well as the number of trials and capacity remaining in the block, were displayed on the left of the screen throughout the experiment.

**Figure 2:**
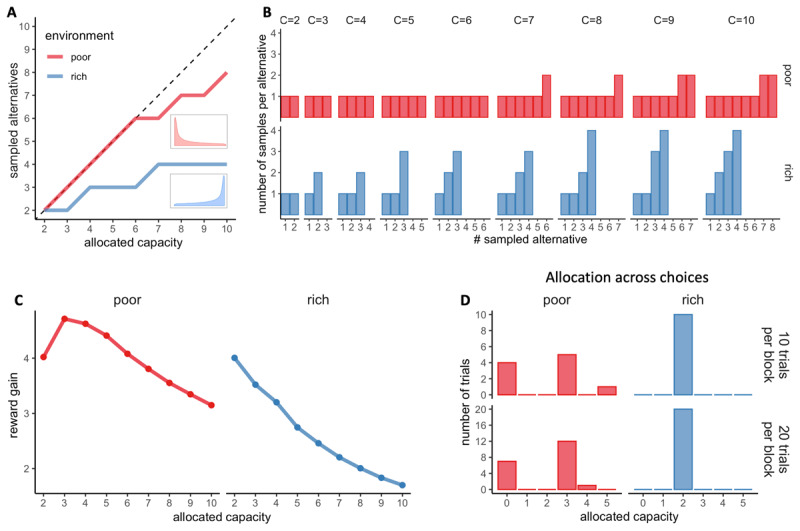
**Optimal allocations of search capacity. A**. Optimal BD trade-offs: number of alternatives sampled (*M*) maximising the expected reward depending on the capacity allocated in the trial and the environment richness (colours). Dashed lines indicate unit slope line. Prior distributions of success (proportion of good quality apricots) of each environment are plotted next to each curve. **B.** Number of samples allocated to each sampled alternative maximising the expected reward (optimal), depending on the capacity allocated *C* and the environment richness (colours). **C.** Reward gain (rg) as a function of the allocated capacity for each environment. rg is defined as *rg* = (*𝔼*[*R*│*optimalmodel, C*] – 𝔼[*R*│*randommodel*])/*C*, where *R* stands for the obtained reward. The reward gain is therefore the difference in reward expected when sampling with capacities from 2 to 10 (assuming an optimal BD trade-off) and when choosing randomly divided by the allocated capacity; such a quantity stresses the importance of following an optimal allocation strategy. **D.** Optimal distribution of trials depending on their sampling capacity, the block size and the environment richness for a capacity ratio *r* of 2.

Independently in each trial and for each supplier *i*, the fraction *p_i_* of good apricots was randomly drawn from a beta distribution (with parameters *μ, ν*). We considered two different environments, varying in the relative abundance of good apricots (*p_i_*), denoted poor (*μ* = 1/3, *ν* = 1) and rich (*μ* = 1, *ν* = 1/3) (prior means in the poor environment: .25, and in rich: .75, see Figure 2A). Participants were either presented to one or the other (but never to both) and they were verbally instructed about the relative richness of the environment they are in (poor/rich: “a majority of suppliers have a low/high proportion of good quality apricots”). Participants are aware that even though alternatives are different in each trial, they are extracted from the same environment. Providing participants with information about the prior distributions is pertinent, as the optimal model presupposes knowledge of the parameters *μ* and *ν*.

Participants were presented with 18 blocks (10 or 20 consecutive purchases with a fixed limited budget), composed of 3 repetitions of each experimental condition (capacity ratio and block length). Additionally, they were first presented with a practice block of 10 trials and a capacity ratio of 2 (these trials were excluded from the analyses). The whole experiment was self-paced, and opportunities were given to participants to rest after each block.

## Allocation of resources within trials: The Breadth-Depth trade-off

### Defining the sampling strategies

Our objective is to investigate how humans allocate limited search capacity over a series of choices to gather information about alternatives whose probability of success is unknown a priori. The sampling strategy covers three levels of behaviour. First, it is defined by how much capacity (number of samples, or coins) is allocated in each choice (trial). Second, it is characterised by how many alternatives are sampled (*M*) in a trial depending on the allocated capacity (*C*). The relation between *M* and *C* defines the BD trade-off where a ratio *M*/*C* of 1 indicates a pure breadth, whereas lower ratios represent strategies leaning toward depth. Finally, the sampling strategy is also characterised by the way samples are allocated within each alternative. We first describe the allocation of resources within a trial, meaning the optimal BD trade-off and optimal resource allocation within the sampled alternatives (see also Moreno-Bote et al., 2020 and Vidal et al., 2022) and then introduce the optimal distribution of resources amongst the consecutive choices.

### Optimal sampling strategy at the alternative level (BD trade-off)

The optimal sampling strategy is based on the work of Moreno-Bote and colleagues (2020), and we present the detailed information here. The framework assumes normative agents who do not exhibit any memory leakage and have knowledge of the environment priors (*μ* and *ν*). Specifically, normative agents aim to maximise expected reward and select the sampled alternatives that maximise the normative value \[{{V}_{i}}~=~\frac{\left({\sum}_{1}^{s}{{O}_{s,i}}~+~\mu \right)}{{{N}_{i}}~+~\mu ~+~\nu}\], where *O*_*s,i*_ represents the outcome of each sample *S* (1 or 0) allocated to alternative *i* drawn from the binomial distribution *B*(*N*_*i*_,*p_i_*), *N*_*i*_ is the total number of allocated samples, and *μ* and *ν* are parameters that describe the beta distribution from which rewards in the environment are drawn. As the actual outcomes are unknown before the capacity is allocated, participants must compute the expectation of the maximum value of all sampled alternatives averaged over all possible outcomes given environment parameters in order to determine the optimal allocation strategy.

The normative strategy is described at two levels, depending on the allocated resources (capacity *C*) and the richness of the environment. Firstly, at the trial level, it predicts the number of alternatives that should be sampled (BD trade-off, see Figure 2A). In a low-capacity scenario (e.g., *C* < 6 for a poor environment), the optimal allocator model predicts pure breadth, meaning that each resource sample should be allocated to a different alternative. As the capacity increases, a sudden change of strategy is observed, with the optimal number of sampled alternatives being approximately a power law function of the capacity (Moreno-Bote et al., 2020). Intuitively, when the agent has more resources, it is better to focus the samples on a few alternatives rather than spreading them across too many, as the latter approach would result in minimal discriminability between the quality of the sampled alternatives. The capacity at which the transition between pure breadth and the BD trade-off occurs depends directly on the richness of the environment. The poorer the environment, the later (at higher capacity) the transition will occur.

In the current experimental paradigm, participants determine the number of samples (capacity) to allocate in each trial. Consequently, at the individual level, we may have a limited number of trials or even none for certain capacities. To overcome this limitation and minimise potential noise, we chose to include in the analysis only capacities for which there were at least three observations (trials) from at least three participants when modelling the BD trade-off. This “rule of three” ensured a minimum level of precision in the measurements and was applied to all analyses and data visualisations, as required.

Secondly, the normative model predicts how many samples should be allocated to each of the sampled alternatives (Figure 2B). Indeed, for a given capacity *C* and number of alternatives sampled *M*, several resource allocations may coexist. For example, a capacity of four (*C* = 4) allocated to two alternatives (*M* = 2) may result in the allocation of 2 samples in two different alternatives each: {2,2}, or the allocation of 3 samples in the first alternative and 1 sample in the second: {3,1}. Visually, participants’ resource allocation, in accordance with previous findings (Vidal et al., 2022), seems to favour homogeneous allocations of resources among the sampled alternatives (e.g., {2,2}) (Figures 8 and S9). However, particularly in deep allocations and rich environments, optimal behaviour involves non-homogeneous allocations of samples to break ties (see Moreno-Bote et al., 2020 and Figures). To ascertain whether participants’ allocations differ from the optimal ones, we used the same method as previously described by Vidal and colleagues (2022), consisting of computing the standard deviation of each sample allocation and comparing it to the standard deviation of the optimal allocation (predicted by Moreno-Bote et al., 2020) using a Wilcoxon test. We found significant deviations from optimality. To evaluate their potential effect on participants’ performance in the task, we compared the observed outcomes (number of high-quality apricots purchased) with those obtained when following the ideal allocator strategy using a t-test. Since we were interested in deviations towards a more homogeneous sampling, we considered only trials where a pure breadth strategy is not optimal (*M*_*opt*_ < *C*) (as in Vidal et al., 2022). However, in the poor environment, this condition is only satisfied when the allocated capacity is equal to or greater than seven, which corresponds to very few trials (166 in total). Therefore, we decided to focus our analysis on the rich environment, where we have sufficient data that satisfy this condition (3545 trials with *C* ∈ [3,10]).

### Method to compare models of Breadth-Depth trade-offs

As in Vidal and colleagues (2022), we explored individuals’ sampling strategies by fitting the number of alternatives sampled *M* as a function of the capacity allocated *C* with three models, separately for each participant and block length experimental condition:

1. A piece-wise power-law model (*W*): \[M(C)=\left\{\begin{array}{c} {{C}^{{{a}_{1}}}}~if~C~\le ~B \\ {{C}^{{{a}_{2}}}}+~b~if~C\ge B \\\end{array}\right\}\], where *B* corresponds to the breakpoint with *B* ∈ {3,4,5, …, *B*_*max*_} with *B*_*max*_ corresponding to the maximum capacity allocated minus one.
2. A linear model (*L*): *M*(*C*) = *aC*.
3. A power-law model (*P*): *M*(*C*) = *C*^a^.

Linear and power-law models were compared using a paired Wilcoxon test on individual R-squared adjusted while power-law and piece-wise power law models were compared using ANOVA (with *α* = .05) at the participant and block length levels. The power-law model captured the empirical relationship between *C* and *M* best in both environments.

Given the results of the model comparisons, the effect of the environment on participants’ sampling strategies was therefore tested using the power-law model. We compared the power factor *α* extracted from power-law fits in each environment (rich and poor) using a Wilcoxon test, and in each block length (10 or 20 trials) using a permutation ANOVA with both block length and environment as factors. This analysis could not be used to compared BD trade-offs between capacity ratio conditions (*r*) as the range of capacities allocated differed greatly depending on *r*. Instead, we tested *M* depending on the capacity allocated, the ratio and the environment using a permutation test ANOVA.

Participants’ BD trade-off were also compared to optimal (Moreno-Bote et al., 2020) by fitting the optimal values of alternatives sampled (*M*) depending on the capacity allocated for each participants (as participants used difference ranges of allocated capacity within trials), using the power law model (see Eq. 3). The power exponents obtained from fitting observed and optimal *M* were then compared with an appropriate test (t-test or Wilcoxon test) within each environment.

### Humans follows optimal and stable BD trade-offs

**Figure 3:**
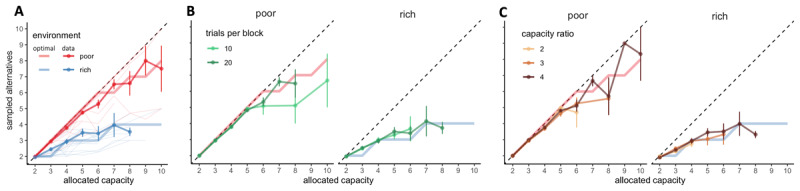
**BD trade-offs are close-to-optimal and adapt to the environment, while being stable among blocks with different length and capacity ratio. A**. Number of alternatives sampled (M) depending on the capacity allocated and the environment richness (colours). Group average and s.e.m. are plotted above individual data (thin light lines) and optimal values of M (thick light lines). Dashed lines indicate unit slope line. **B-C.** Colours represent the block length (B) or the capacity ratio *r* (C). N = 20 per environment.

First, we focused our analysis on the *BD* trade-off, which refers, in each trial, to the number of alternatives sampled (*M*) as a function of the capacity allocated within the trial (*C*). Allocating few samples to many different suppliers would be considered as *Breadth* behaviour (for example, when *M* = 6 suppliers are sampled with a total *C* = 6 coins), whilst allocating many samples in few suppliers is considered as *Depth* (for example, when *M* = 1 supplier is sample with *C* = 6 coins). In line with previous theoretical (Moreno-Bote et al., 2020) and experimental findings (Vidal et al., 2022), we observed that the number of alternatives sampled varied with both the capacity allocated and the environment richness in close-to-optimal manners (Figure 3). At lower capacities, participants sampled as many alternatives as possible, following a pure-breadth strategy. As more capacity *C* was allocated, participants tended to focus more capacity on few alternatives. As previously reported (Vidal et al., 2022), this BD trade-off was best captured using the power model \[\left(R_{adj}^{2}~=~.97~\pm ~.03\right)\] than a linear model \[\left(R_{adj}^{2}~=~.96~\pm ~.04\right)\] (paired Wilcoxon test, *V* = 112, *p* = .0016) and the optimal piece-wise power law model (all ANOVAs not significant, for *α* < .05). We also replicated previous findings (Vidal et al., 2022) showing that environment richness promotes depth over breadth (power law exponents: *a*_*poor*_ = .95 ± .08, *a*_*rich*_ = .76 ± .15, see Methods for models details) (Wilcoxon test, *W* = 61, *p* = 7.94 × 10^–5^, Figure 3A). We compared these exponents with the ones obtained by fitting the optimal values of *M* with the power-law model (poor: .99 ± .02, rich: .73 ± .03) and didn’t find any significant difference in the poor (paired Wilcoxon test, *V* = 47, *p* = .10) or in the rich environment (paired t-test, *t*_19_ = .90, *p* = .38), suggesting that overall, participants allocated capacity amongst an optimal number of alternatives. These findings differ to previously published results, using a design where the number of samples to be allocated per trial was fixed, showing that people are slightly sub-optimal at balancing breadth and depth (Vidal et al., 2022). Even though, sampling strategies from both studies cannot be formally compared due to different capacities used, they raise the idea that more freedom in search strategy may stimulate performance. We come back to this point in the discussion.

Even though environment richness had a clear impact on the participants’ BD trade-off, initial capacity of the block (*N_c_*) did not. Indeed, on one hand, block length did not have a significant effect on the power exponents (permutation ANOVA, *p* = .94) and neither interacted with the environment (*p* = .59, Figure 3B, *a*_*poor,*__10_ = .95 ± .09, *a*_*poor,*__20_ = .95 ± .08, *a*_*rich,*__10_ = .78 ± .16, *a*_*rich,*__20_ = .75 ± .15). The difference between the power exponents in the short and long block was significantly smaller than .1 (paired TOST test, *t*_39_ = –5.30, *p* = 2.43 ×10^–6^), and therefore negligible. On the other hand, capacity ratio *r* did not have a significant effect on *M* (permutation ANOVA, *p* = .60, Figure 3C), neither interactions between *r* and allocated capacity *C* (*p* = .93), or between *r* and environment richness (*p* = .95) were significant. These results suggest that participants follow a balance between breadth and depth adapted to the environment but stable across capacity available and the horizon, meaning that the allocation strategy exclusively depends on the number of allocated samples on each trial and the environment richness.

## Allocation of resources across trials: Exploring behavioural fluctuations

**Figure 4:**
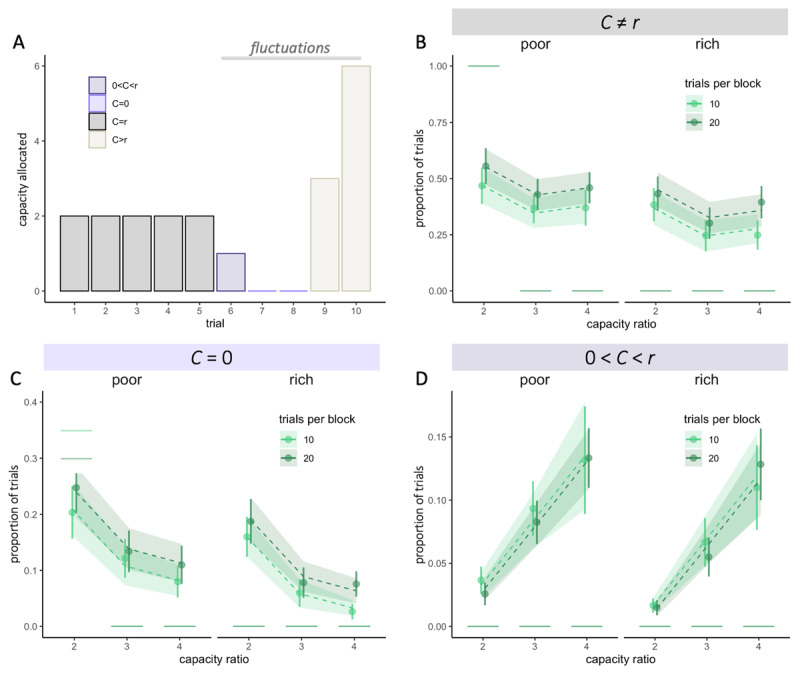
**Participants capacity allocation fluctuates more when little capacity is available and with larger horizons**. **A**. Example of capacity allocation (*C*) from one participant throughout consecutive trials in a block of length 10 and ratio *r =* 2. **B-D**. Fluctuations are defined as trials for which the allocated capacity *C* is different from *r*. Observed averaged proportions of trials (dots) where *C ≠ r* (fluctuations – B), *C* = 0 (skipped trials – C) and 0 < *C* < *r* (D), depending on the capacity ratio *r*, block length (colours) and the environment (poor or rich). Vertical bars represent s.e.m. of the data, while dashed lines and shaded areas represent respectively the predicted averages and s.e.m. using Linear Mixed Effect Models (LMEM). Horizontal segments represent the proportion of each type of trials predicted by the optimal model.

We considered variations in resource allocations (fluctuations), trials where the number of allocated resources differed from the capacity ratio of the block (*C* ≠ *r*) (see Figure 4A). Among these variations, we identified three categories: trials with no capacity allocated (*C* = 0), referred to as “skipped trials”, trials with a non-null capacity allocated below the capacity ratio (0 *< C* < *r*), and trials with a capacity allocated above the capacity ratio (*C* > *r*).

### Optimal sampling strategy at the choice level

To establish how capacity should be optimally allocated amongst the choices (purchases or trials), we calculated the reward gain, that is the expected reward gain defined as the difference between the expected reward obtained during the purchase phase when sampling with a capacity *C* (using Monte-Carlo simulations and assuming an optimal allocation of samples within the alternatives – see section above) compared when selecting an alternative randomly (average prior distribution of the environment). We divided this value by *C* to assess the unit reward gain per sample and infer the optimal behaviour. In the rich environment, we observe that the unit reward gain is maximised when sampling with a capacity of 2 and decreases with higher capacities (Figure 2C – right panel). In contrast, in the poor environment, it is maximised for a capacity of 3 and is higher for a capacity of 4 and 5 compared to 2 (Figure 2C – left panel). As a result, while in the rich environment, it is optimal to always allocate a capacity equal to the capacity ratio (Figure 2D – right panels), in the poor environment, fluctuations in the resource allocation are optimal for low-capacity ratio (*r* = 2) (Figure 2D – left panels). Indeed, it is therefore optimal to allocate 3 coins a majority of times while making sure not to be left with 1 or 2 coins which are associated with low reward gains. If this is the case it is then better to anticipate and keep the remaining coins in order to allocate 4 or 5 samples per trial. The remaining trials are skipped, meaning no capacity is allocated. Following this optimal allocation is associated with an average reward of 34.3 in blocks with 10 trials and 34.4 in blocks with 20 trials, while a homogenous allocation across choices is rewarded on average 33.0.

### Method to measure behavioural fluctuations

To test these predictions that fluctuations should occur only in the poor environment with a capacity ratio of two, we first calculated the proportions of trials belonging to each category of fluctuations within each individual block, then averaged them across blocks, and finally averaged them across participants. We examined the impact of our factors of interest (ratio, block length, environment richness) on the occurrence of these fluctuations using linear mixed-effects models. Participants were included as random intercepts in the models.

We primarily selected conditions in which it was optimal *not* to fluctuate (r = {3,4} in the poor environment and all conditions in the rich environment), allowing us to observe whether participants would still display fluctuations in cases where it would be suboptimal according to a reward-maximisation framework. This approach enables us to explore potential alternative motives behind these fluctuations, beyond immediate reward maximisation (see bellow “*Extension of the ideal allocator model*”). To assess this, we calculated the proportion of trials in each fluctuation category within individual blocks, averaged them across blocks, and then averaged them across participants. We examined the effects of our factors of interest (ratio, block length, and environment richness) on the occurrence of fluctuations using linear mixed-effects models, with participants included as random intercepts.

For completeness we also described fluctuations in the resources allocation using two other methods. First, we computed the coefficients of variation (*CV*) of the sample allocation within each block, given by:


\[CV=std\left(\vec{P}\right)/r,\quad  {\rm with}\ \vec{P}=\left\{{p}_{0},\ {p}_{2},\ {p}_{3},\ \ldots,\ {p}_{10} \right\}\]

Where *p*_*c*_ represents the probability to allocate a capacity *c* inside a trial and *r* the capacity ratio of the block. Results are presented in the supplementary Figure S1 and reveal similar effects as when considering fluctuations as trials with an allocated capacity *C* different from the capacity ratio *r*.

Secondly, we computed the entropy *H* of participants capacity allocations within each block, given by:


\[H=-{{{\sum}^{}}_{c}}{{p}_{c}}\cdot \log {{p}_{c}}\]

This measure does not control for the amount of resources available, but still reveal that fluctuations are larger in the longer compared to shorter blocks (\[\chi _{1}^{2}~=~43.91\], *p* = 3.44 × 10^–11^). Results are reported in the supplementary Figure S2.

### Humans’ allocation capacity fluctuates more than optimal

Overall, we found larger fluctuations than what would be predicted by an optimal allocation strategy (mean ± sd proportion of trial with *C* ≠ *r*: .40 ± .28, Wilcoxon test, *V* = 694.5, *p* = 2.21 × 10^–15^, Figure 4B).

We observed that these fluctuations corresponded to two strategies: skipping a trial (no resources allocated, Figure 4A – light purple) or spending a capacity inferior to the ratio, 0 < C < *r*, defined as ‘under-sampling’ (Figure 4A – dark purple). While the first was predicted by the optimal allocator (at least in poor environments with *r* = 2), the latter wasn’t. In line with the optimal strategy, we found that when available resources are scarce (low *r*), allocation fluctuates more from trial to trial. This leads to more skipped trials than in higher capacity ratio conditions (*F* = 46.07, *p* < 4.2 × 10^–8^, Figure 4C), a strategy that allows more extensive search on the remaining trials. However, contrary to what predicted by the optimal model, we didn’t observe significantly more skipped trials in the poor compared to the rich environment for block with a capacity ratio of two (poor: 23.3 ± 19.9%, rich: 17.8 ± 16.7%, *W* = 168, *p* = .39, Figure 4C). Finally, fluctuations are amplified in longer blocks (*V* = 4257, *p* = 2.13 × 10^–15^, Figure 4C – colours), which had a significantly higher proportion of skipped trials than optimal, suggesting that participants might capitalise on the larger available resources to increase exploration.

Participants also chose to allocate some resources but less than the capacity ratio on a significant number of trials (7.46 ± 7.13%, *V* = 780, *p* = 5.44 × 10^–8^, Figure 4D), which was not predicted by the optimal model. These fluctuations increase with the capacity ratio (LMEM, *F*_2_ = 45.19, *p* < 2 × 10^–16^) but were not affected by the block length (*F*_1_ = .08, *p* = .78) and the environment richness (*F*_1_ = .68, *p* = .42). Such fluctuations may reflect a balance between selectively gathering more information in some trials (*C > r*) whilst trying to avoid skipping trials (and leaving the final choice to chance).

Overall, these deviations from optimality had little impact on performance (mean ± sd: –3.07 ± 4.81%), but the more the proportion of fluctuations diverged from the one predicted by the optimal model and the greater was the loss in outcome. Indeed, our observations indicate that in the poor environment, the relationship between participants’ average outcomes and their level of fluctuations (trials with *C* ≠ *r*) was best captured by a second-order polynomial regression (\[R_{adj}^{2}~=~.26,~p~=~.029\]) as opposed to a linear regression (\[R_{adj}^{2}~=~.12,~p~=~.078\]) (Figure S4 – left). In fact, participants achieved highest average outcomes when their resource allocation among choices approximated the predicted optimal percentage of fluctuations (33.3% of trials overall as optimal fluctuations are 100% when r = 2 and 0% when r = 3 or 4; see Figure 2D and Figure S4 – dotted lines). In the rich environment, as predicted, the association between participants’ average outcomes and fluctuations was more accurately captured by a linear regression (\[R_{adj}^{2}\] = .15, *p* = .053), than by a second-order polynomial regression (\[R_{adj}^{2}\] = .12, *p* = .13) (Figure S4 – right). In this case, higher outcomes were achieved when fluctuations were lowest (at 0). This finding confirmed that a more uniform allocation of resources among choices tended to produce higher outcomes in rich environments. In order to obtain robust estimations of the mean outcomes received, this analysis was performed by averaging data over all the trials regardless of the capacity ratio condition. Results confirm that the experimental design influenced participants’ outcomes as anticipated, potentially shaping their strategies to align with the ideal allocator model.

### Sequential effects observed in the allocation of resources

#### Evidence for an intentional strategy

We delved deeper into the potential constraints and objectives associated with participants’ behaviour in allocating little capacity (capacity *C* inferior to the capacity ratio *r*, referred as *C < r*) in a trial, resulting in fluctuations in resource allocation. Specifically, we calculated the capacity allocated in a given trial based on its relative position to the nearest trials with *C < r*. This analysis aimed to investigate whether these trials are a product of anticipatory strategies or rather an adaptation after having previously over-allocated the available capacity.

**Figure 5:**
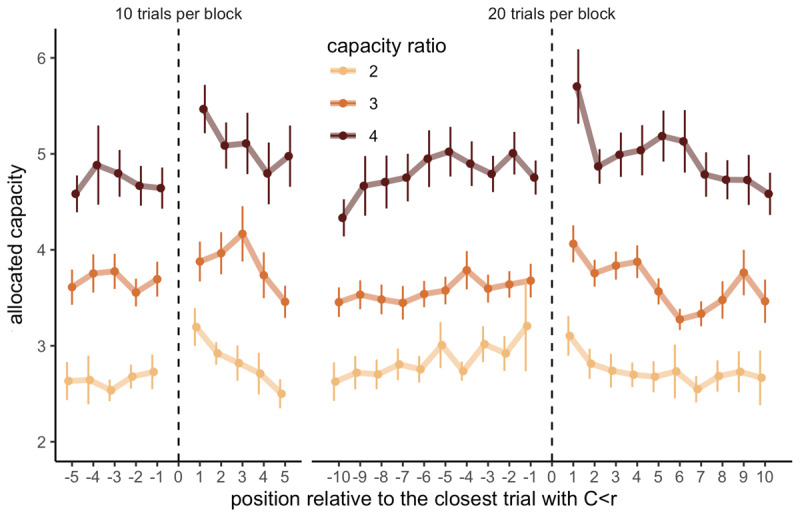
**Allocating fewer capacity than the ratio is part of an anticipated strategy for sampling more in the next trial**. Capacity allocated in trials depending on their relative position to the closest trial with an allocated capacity inferior to the capacity ratio of the block (*C < r*), depending on the block length (left: 10 trials, right: 20 trials) and the capacity ratio (colours). Each data point represents the average of 12 to 37 participants with at least 3 data points per participant. Vertical bars represent s.e.m.

Indeed, participants allocated significantly more resources in trials directly following trials with low capacity allocations (e.g., fewer capacity than the ratio; *C < r*) compared to trials directly preceding them (LMEM, \[\chi _{1}^{2}\] = 16.24, *p* = 5.58 × 10^–5^, Figure 5), suggesting that overall, allocating little capacity is part of an anticipatory strategy to explore further later. This effect didn’t interact with either the capacity ratio (\[\chi _{2}^{2}\] = .69, *p* = .71), nor the block length (\[\chi _{1}^{2}\] = .81, *p* = .37).

We then investigated the origin of the observed fluctuations beyond those predicted. One possibility is that they resulted from an exhaustion of resources given the allocation policy over the initial part of the block, leading participants to obligatorily skip or underspend in the remaining last trials. Another possibility is that the decision of skipping trials was intentional, meaning that they had enough resources to sample the trial but decided not to do so. We observed that the great majority of trials with no allocated capacity (skipped) (mean ± s.d. across participants: 82 ± 31% and overall: 84%) occurred while some capacity is remaining (*N_r_* ≠ 0), and therefore intentionally, suggesting the possibility that participants may be saving resources for later.

#### Participants adapt their sampling strategy to the choice outcome

**Figure 6:**
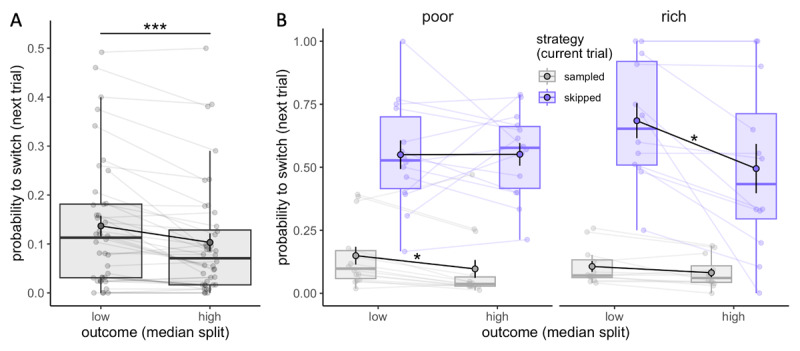
**Participants adapt their sampling strategy to the outcome received. A-B**. Probability to switch strategy in the next trial (from sampling to skipping and vice versa) depending on the outcome received (median split, calculated separately for individuals and skipped and sampled trials, N = 40) (A) and the strategy in the current trial (sampling or skipping) as well as the environment (poor: N = 14, rich: N = 12) (B). Boxplots represent the 1^st^ and 3^rd^ quartiles of the data distribution, with thicker horizontal black lines corresponding to medians and whiskers extended to the largest value no further than 1.5 times the inter-quartile range (IQR). Lighter dots represent individual data and are connected with lighter lines, while group averages are plotted on top (colour dots circled in black) and connected by black lines. Vertical black bars represent s.e.m. ‘*’: *p*_*adj*_ < .05, ‘***’: *p* < .001.

In the earlier analysis we observed that participants adapted their sampling strategy to the limited capacity available, and prioritised spending little to no capacity in order to spend more later (Figure 5). Next, we addressed whether participants also adapted their strategy according to the outcome received in a given trial. To explore that, we divided trials as a function of the outcome received (median split), separately for sampled (*C* > 0) and skipped trials (*C* = 0), where the outcome is either received following the selection of one of the sampled suppliers in a sampled trial or a randomly selected supplier in a skipped trial. Based on this categorisation, from one trial to the next, participants could either maintain the same strategy (sample-sample or skip-skip) or switch their strategy (sample-skip or skip-sample). Our investigation focused on how the magnitude of the outcome received in a given trial influenced the probability of repeating or, conversely, switching the strategy in the next trial. These analyses exclusively considered trials in which some capacity was remaining (*N_r_* ≠ 0), ensuring that both behaviours (sampling or skipping) were feasible. The results revealed that participants are more likely to switch strategy (sampling after skipping, or vice versa) following low outcome trials (.14 ± .13) compared to high outcome ones (.10 ± .12) (Wilcoxon test, *V* = 504, *p* = 6.76 × 10^–5^, Figure 6A). The magnitude of this effect was not affected by the trial type (sampled or skipped, *F* = 1.78, *p* = .20) nor the environment richness (permutation ANOVA, *F* = 3.85, *p* = .061), although we found a significant interaction between the environment richness and trial type (*F* = 6.91, *p* = .012). Post-hoc analyses revealed that the higher probability to switch strategy is only found following sampled trials in the poor environment (*V* = 93, *p*_*adj*_ = .034; after skipped trial: *t*_13_ = –.03, *p*_*adj*_ = 1, Figure 6B – left), while in the rich environment this effect is found solely after skipped trials (*t*_11_ = 2.17, *p*_*adj*_ = .042, after sampled trial: *V* = 66, *p*_*adj*_ = .14, Figure 6B – right). Although these results have to be interpreted with caution due to the limited sample size included, they suggest that participants consider differently the outcome received depending on the environment richness. In the rich environment, high outcomes are easily obtained and expected even when choosing randomly a supplier (skipped trial), therefore receiving a low outcome may be more surprising and enforce a switch in strategy for sampling. In contrast, in the poor environment, low outcomes are expected when skipping and it’s not surprising that they don’t affect participants’ future strategy.

## Behavioural fluctuations beyond mere reward maximisation

### Extension of the ideal allocator model of resources across trials

We observed that participants exhibited more fluctuations in their resource allocations compared to what is optimal. To account for this phenomenon, we extended the optimal model to predict and explain the increased occurrence of these fluctuations. Using this computational tool provides a structured framework for disentangling the complex influences on behaviour, offering insights into why individuals depart from purely reward-maximising strategies. Indeed, the variations in resource allocations allow for the sampling of certain trials with a higher frequency, enabling participants to gather more precise information about the quality of the alternatives. We proposed that one motivation behind these fluctuations is to ensure informative sampling, which means that sampling informs the selection of a better alternative (by avoiding ties for example). Another motivation could be to explore the amount of information and outcomes obtained by using different numbers of resources, contributing to a broader learning process of the environment. Additionally, we noticed substantial variability in participants’ tendency not to allocate any resources in a trial (skipping). Randomly selecting a supplier for the final purchase may indeed be associated with increased risk and explain why some participants try to avoid such situations.

Consequently, we proposed a model where the expected utility (*EU*) to maximise is a weighted sum of different factors (Eq. 1). These factors include the expected reward *R*_*c*_ (following the optimal allocation of C samples), the information benefit *I*_*c*_ (weighted by a factor *α*), a penalty for skipping trials weighted by a factor *β*, and an entropy bonus weighted by a factor *γ*. The probability *p*_*c*_ represents the likelihood of using a capacity C in the block.


Eq.1 \[EU={\sum}_{c=0}^{10}{{R}_{c}}{{p}_{c}}+\alpha {\sum}_{c=0}^{10}{{I}_{c}}{{p}_{c}}-\beta {{p}_{0}}+\gamma \left(-{\sum}_{c=0}^{10}{{p}_{c}}\log {{p}_{c}} \right)\]

Here and below we don’t write explicitly the dependence of *EU* and other variables on the parameters of the beta distribution *μ* and *ν* to simplify the notation.

For computational convenience, we considered capacities ranging from 0 to 10. Capacities exceeding 10 were excluded as their probability of occurrence in a trial is only 0.31%, and they are present in only 3.89% of the blocks.

Regarding the information benefit *I*_*c*_, we modelled it in three different ways and estimated them using Monte-Carlo simulations (N = 100 000 simulations).

1.
\[{{I}_{max,C}}~=~E\left[ma{{x}_{i}}\left({{S}_{i,C}}\right)~|~\mu,\nu,C\right]\]

  Where *i* runs over the alternatives sampled according to the optimal allocator, in a given environment, determined by the parameters *µ* and *ν* of the beta distribution, and with a given allocated capacity *C*.
  We proposed that an informative sampling may be represented by the expected highest sampling probability *S*_*i,C*_, with \[{{S}_{i,C}}~=~\frac{{\sum}_{1}^{{{N}_{i}}}{{O}_{s,i}}}{{{N}_{i}}}\]. *O*_*s,i*_ represents the outcome of each sample *S* (1 or 0) allocated to alternative *i* and *N*_*i*_ is the total number of samples allocated to the alternative *i*.
  This measure does not assume prior knowledge of the environments’ prior distribution (*μ, ν*). We hypothesised that such information benefit may be particularly valuable to guide the selection of a ‘good’ alternative in the poor environment where getting positive sample outcome (*O*_*s,i*_) is very unlikely.
2.
\[{{I}_{single,C}}~=~P\left({{A}_{\max \left({{S}_{i,C}}\right)}}~=~1|\mu,~\nu,~C\right)\]

  Where *A*_*max*_(*S*_*i,C*_) stands for the number of alternatives with *S*_*i,C*_ = max_*i*_(*S*_*i,C*_).
  The information benefit corresponds here to the probability of having a single alternative *i* with the highest sampling probability *S*_*i,C*_. We hypothesised that, especially in the rich environment where receiving positive sampling outcome is very frequent, being able to distinguish the best sampled alternative (break ties) may drive participants sampling strategy.
3.
\[{{I}_{entropy,C}}~=~-{\sum}_{C=0}^{10}~{{p}_{{{S}_{i,C}}}}\log {{p}_{{{S}_{i,C}}}}\]

  Finally, we proposed the information benefit to corresponds to Shannon’s entropy (Rényi, 1961), as it is a common measure used to quantify information content. \[{{p}_{{{S}_{i,C}}}}\] represents here the probability distribution of the sampling probabilities *S*_*i,C*_ for a given alternative *i* and allocated capacity *C*.

We observe, in both environments, that the evolution of the information benefits as a function of the allocated capacity differs from the one observed in the expected outcome, suggesting its possible impact on participants’ sampling strategy, independently of the reward (Figure S3).

An important limitation shared by all these information benefits is to be estimated based on an optimal allocation of samples within each capacity and environment. Results showed that participants closely follow optimal BD trade-offs in both environments (see Figure 3A) but within each alternative, the allocation of samples differs from the one maximising the reward, especially in the rich environment where participants have been shown to favour homogenous samples allocations (Figure 8).

We observed that in both the poor and rich environment, the model predicting the best the data was the one including *I*_*single,C*_ (2) so we further reported models with *I*_*C*_ = *I*_*single,C*_.

### Method for fitting the extended ideal allocator model

The underlying assumption of the fitting procedure consists in considering that the participants’ behaviour arises from the optimisation of the expected utility *EU* given in Eq. 1. In other words, we assume that participants behave optimally with respect to a fixed set of parameters *α, β* and *γ*. Therefore, we first computed the optimal probabilities (that is, the one maximising *EU*) for a generic configuration of *α, β* and *γ*. *p*_*c*_ representing the probabilities of allocating a capacity *C* in a given trial within a block, they have to meet the following constraints:

1.
\[0~\le ~{{p}_{c}}~\le ~1\]

2.
\[\Sigma _{c=0}^{10}~{{p}_{c}}~=~1\]

3. \[{\sum}_{c=0}^{10}~{{p}_{c}}~\cdot C~=~r\], where *r* represents the capacity ratio of the block.

This constrain entails that all the resources initially available in a block (*N_c_*) are allocated. In the data, a significant number of blocks didn’t comply with this constraint (135 blocks, 19.5%), however a great majority of them had only one sample not allocated (78 out 135). As a result, we chose to be more flexible and only excluded blocks for which less than the initial capacity minus one had been allocated (corresponding to 57 blocks, 8.23%).

To find the optimal probabilities *p*_*c*_ given these constraints we used the Lagrangian multiplier method. We looked then for the critical points of the following Lagrangian function:


Eq.2 \[\mathcal{L}~=~EU~+~{{\lambda}_{1}}~\left(\underset{c}{{{\sum}^{}}}\,{{p}_{c}}~-~1\right)~+~{{\lambda}_{2}}~\left(\underset{c}{{{\sum}^{}}}\,{{p}_{c}}~\cdot C-r\right)\]

obtaining


Eq.3 \[p_{c}^{*}~=~{{e}^{\frac{1}{\gamma}\left({{R}_{c}}~+~\alpha {{I}_{c}}~-~\beta {{\delta}_{0,c}}~+~{{\lambda}_{1}}~+~c{{\lambda}_{2}}\right)~-~1}}\]

where δ_0,c_ is the Kronecker delta and with λ_1,2_ given by imposing


Eq.4 \[\frac{\partial \mathcal{L}}{\partial {{\lambda}_{1,2}}}~=~0\]

and


Eq.5 \[\frac{\partial \mathcal{L}}{\partial {{p}_{c}}}~=~0\]

Note that the constraint (1) is automatically satisfied by the solution of Eq.3 and by satisfying the constraint (2).

A generic closed form solution for the Lagrange multipliers cannot be found (Abel-Ruffini theorem, not shown) and a numerical method is implemented to invert the equations for λ_1,2_ (using the function *fsolve* in Python).

In such a way, the optimal probabilities can be computed per each *α, β* and *γ*. To fit these parameters, we minimised the sum S of the Euclidian distance between the observed probabilities *p*_*c*_and the optimal predicted ones \[p_{c}^{*}\] and a penalty accounting for the size of *α, β, γ*, such as:


Eq.6 \[S~=~|{{p}_{c}}~-~p_{c}^{*}|~+~\omega \left(|\alpha |~+~|\beta |~+~|\gamma |\right)\]

Where *ω* is a regularisation parameter. Different values of *ω* were tested to achieve a good balance between consistency of the parameters *α, β, γ* fitted and goodness of fit. As a result, a value of *ω* = 1 was selected.

In order to compute criterions taking into account both the goodness of fits and the risk of overfitting (Akaike Information Criterion – AIC and Bayesian Information Criterion – BIC), we also computed the log-likelihood L of the data, given by:


Eq.7 \[L~=~\log \left(\frac{N!}{{{\Pi}_{c}}{{n}_{c}}!}~\Pi p_{c}^{{{*}^{{{n}_{c}}}}}\right)\]

where *n*_*c*_ is the number of times a capacity *C* was allocated in the block, \[p_{c}^{*}\] the optimal probabilities extracted from the model and


Eq.8 \[N~=~{{{\sum}^{}}_{c}}{{n}_{c}}\]

**Figure 7:**
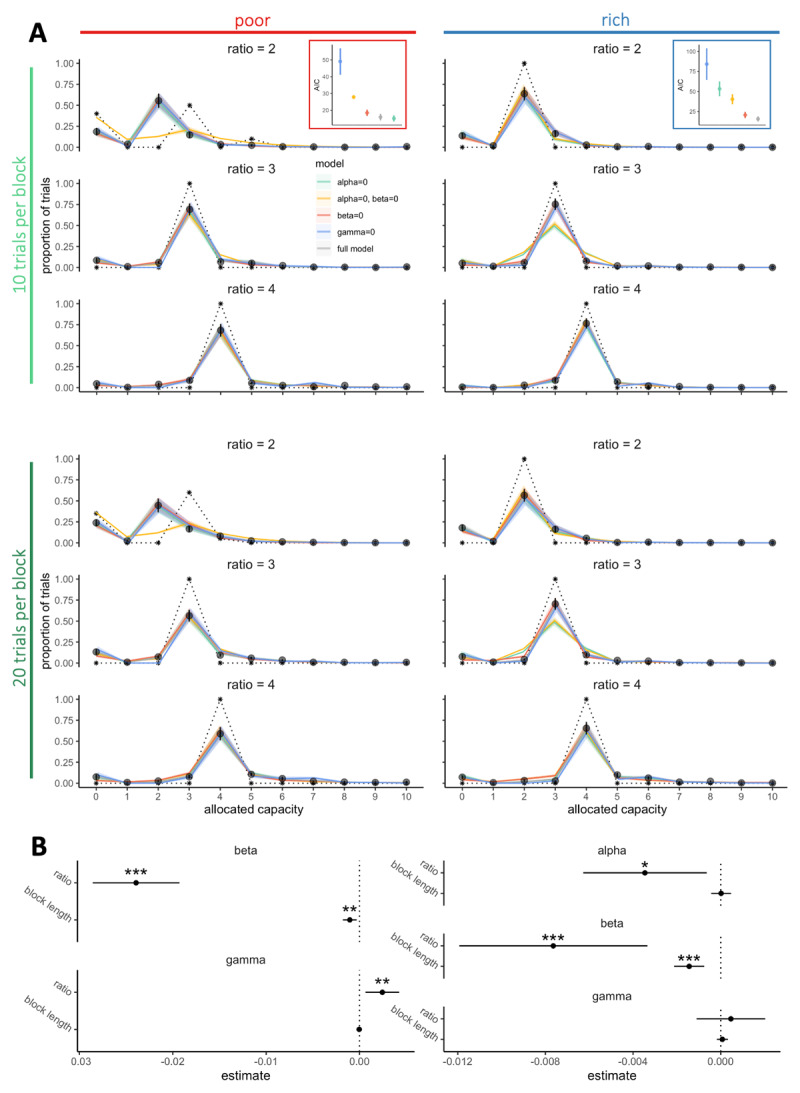
**Participants sampling strategy considers exploration and individual risk aversion features. A**. Probabilities to allocate a capacity *C* from 0 to 10 depending on the environment richness (poor: left panels, rich: right panels) and the block length (10 trials per block: upper panels, 20 trials per block: lower panels). Black points represent the averaged observed probabilities across participants and vertical bars the s.e.m. Colours lines represent the averaged fitted probabilities for each model and the shaded areas the s.e.m. across participants and dashed black lines represent the optimal probabilities for each condition (model maximising only the expected reward). In each environment, the averaged goodness of fits across participants (AIC – Akaike information criterion) were estimated by fitting the data within each block (regardless of block length) using the five different models. Vertical bars represent s.e.m. across participants. **B**. Factors (alpha, beta and gamma) extracted from the model predicting the data the best (‘alpha = 0’ in the poor environment and ‘full model’ in the rich environment) regressed with the capacity ratio and block lengths using LMEM. Estimates represents the extracted coefficients of regressions (bars represents 95% confidence intervals). ‘*’: *p* <. 05, ‘**’: *p* < .01, ‘***’: *p* < .001. N = 20 in each environment.

Model comparison is performed by comparing both AIC (Figure 7A) and BIC (Figure S7) as the latter favours simpler models.

### Identifying deviations from optimality observed in the resource allocation between choices

We have observed that the way participants allocate their available resources amongst the consecutive choices differed greatly from the optimal model. However, the optimal model considers expected reward maximisation as the only goal. To better understand potential origins of participants performance deviations from optimality, we fitted in each block the observed probability of allocating a capacity *C* from 0 to 10, *p*_*c*_ with an extension of the optimal model. In addition to maximising net reward, we included in the model an intrinsic motivation for sampling with higher variability across trials (represented by the entropy term in Eq.1).

Specifically, in addition to the expected reward for each capacity allocated *R*_*c*_, the expected utility EU modelled here incorporates three additional factors that we hypothesised are influencing participants sampling behaviour. First, a factor related to information gain (second term). We based this factor on findings suggesting that not only experienced, but also fictive rewards and valuable information about future outcomes have been found to be encoded in the brain (Bromberg-Martin & Hikosaka, 2009; Bromberg-Martin & Monosov, 2020; Hayden et al., 2009), establishing a framework for curiosity and explorative behaviours aimed at reducing uncertainty about the environment (Gottlieb, 2012). To accommodate this aspect, we introduced an information benefit *I*_*c*_ which can take different forms (see Methods for more details). Here we report models including the best fitting *I*_*c*_, corresponding to the probability of observing a single highest sampled outcome (*S*_*i*_) (see Figure S5). We hypothesise that participants may want to maximise this probability to later facilitate the selection of one out of the many sampled alternatives. Second, we incorporated factors related to individual traits. Individual features such as risk aversion (third term in Eq.1) have been demonstrated to influence the allocation of limited resources in various types of uncertain decisions (Chronopoulos et al., 2011; Dow & Werlang, 1992; Tulloch et al., 2015), suggesting that it might affect participants’ propensity to skip a trial (*p*_0_) and leave it to chance. Finally, participants sampling behaviour may be driven by a tendency to occupy action-state space (maximum occupancy principle), compelling them to try out various resources allocations and gain a global understanding of the environment (Ramírez-Ruiz et al., 2024). To model this, we introduced an entropy term. These three factors in the model were respectively weighted by parameters α, β and γ.

### Participants sampling strategy incorporates exploration, individual risk aversion features, as well as information benefit, but only in rich environments

We fitted, for each block, the probability to spend a certain capacity *C* in a trial with five different models including combinations of the optimal model with the three factors described above (Figure 7A). The results showed that the notable deviations from optimality observed in the empirical data, especially in the probability to skip a trial altogether (Figure S6A) and the probability of low capacity allocation (i.e., 0 < C < *r*) (Figure S6B), are well captured by the full model (including optimised *α, β* and *γ* parameters). We then fitted partial models excluding one of the three parameters (setting either *α, β* or *γ* equal to 0) to evaluate the importance of each of them in predicting the data. Looking at averaged AIC (Figure 7A) and BIC (Figure S7), we observed that in the rich environment, the full model is required to reproduce participants’ sampling behaviour. Instead, in the poor environment a model including only exploration and risk aversion components (*α* = 0, *β* ≠ 0, *γ* ≠ 0) was sufficient to predict the data. Indeed, in the poor environment, the majority of the alternatives are associated with very low outcomes. Better alternatives are therefore easily detectable, which may explain that participants may focus on maximising the reward above and before the sampling information received. In contrast, in the rich environment, good alternatives are easy to find but one’s need to rely on an efficient sampling to select the best alternative. These results are also in line with previous literature showing that the value of information increases when higher stakes are in play (Blanchard et al., 2015). Analyses of goodness-of-fit further demonstrated the significance of exploration in participants’ sampling strategies, as models excluding this component (γ = 0) performed poorly in predicting the data. Moreover, in the poor environment, although models with null values for either information benefit or risk aversion (either α = 0 or β = 0) provided relatively good fits compared to the full model, the model without both (α = β = 0) resulted in significantly worse predictions, particularly concerning the proportion of skipped trials. This finding indicates that, in addition to maximising reward and exploration, participants also adopt a sampling strategy that aims to minimise the uncertainty associated with skipping a trial.

Additionally, among of the three information benefits considered (*I*_*C*_, see Methods), we found that the one providing the best fit to participants’ sampling strategy is the model corresponding to the probability of having a unique sampled alternative with the highest observed sampled probability (number of positive samples out of the total number of samples allocated within this alternative) (*I*_*c*_ = *I*_*single,c*_) (Figure S5). This information is likely to be highly valuable in guiding participants towards selecting one of the sampled alternatives for their final purchase and reducing the uncertainty associated with this choice, particularly in the rich environment where distinguishing the best alternative among many good ones may be more challenging.

Following the initial model selection, we analysed how the factors (α, β and γ) estimated from the best fitting models changed with the experimental variables manipulated (Figure 7B). In both the rich and poor environments, we observed that β decreased with capacity ratio (poor: \[\chi _{1}^{2}\] = 102.01, *p* < 2.2 × 10^–16^, rich: \[\chi _{1}^{2}\] = 12.15, *p* = 4.90 × 10^–4^) and block length (poor: \[\chi _{1}^{2}\] = 7.51, *p* = .0061, rich: \[\chi _{1}^{2}\] = 16.68, *p* = 4.43 × 10^–5^), suggesting the more resources are available and the less participants are reluctant to skip sampling. While it might initially appear counterintuitive, given the lower frequency of skipped trials in blocks with larger capacity ratios, it aligns with the optimal model’s predictions. As the capacity ratio grows, the model suggests that skipping a trial becomes less optimal, leading to an increase in the associated loss in expected reward. Consequently, although participants tend to skip fewer trials when the capacity ratio is larger, the model predicts a diminished level of risk aversion.

Additionally, we found that in the poor environment, where good alternatives are scarce, exploration may be accentuated as more resources are available, as suggested by the increase in γ with the capacity ratio (\[\chi _{1}^{2}\] = 7.06, *p* = .0079). Such increase was not found in the rich environment (\[\chi _{1}^{2}\] = .34, *p* =. 56), nor with the block length in any of the two environments (poor: \[\chi _{1}^{2}\] = .049, *p* = .82, rich: \[\chi _{1}^{2}\] = .36, *p* = .55). Finally, in the rich environment, we observed that α significantly decreases with the capacity ratio (\[\chi _{1}^{2}\] = 5.78, *p* = .016) but not with the block length (\[\chi _{1}^{2}\] = .007, *p* = .93), suggesting that participants look to maximise the information benefit when resource are scarce.

Additionally, as a reality check, we fitted our data with the same models excluding the reward component (*R*_*c*_ = 0). As expected, these models systematically gave worst fits, suggesting that participants are doing the task correctly and try to maximise the received outcomes.

In summary, these results illustrate that the deviations from optimality observed in participants’ sampling behaviour are not a result of random decision processes, but rather stem from systematic heuristics that are integrated into a comprehensive strategy considering both overarching goals and individual characteristics.

### Balancing under-sampling and skipping is explained by estimated level of risk aversion

We further explored the strategies underlying fluctuations at the individual level and whether our extended model captures some of the differences between participants. First, we observed that for participants exhibiting significant fluctuations (at least 15% of the trials), allocating either no capacity or a capacity lower than the capacity ratio (‘under-sampling’: 0 < C < r) in at least one trial per block on average, the more participants skipped sampling and the less they under-sampled (*tau* = –.46, *p* = .006, N = 19) (Figure S8A). To control from spurious correlations computed on the same data set, we randomly split the 18 blocks in two subsets *S* (S = {1,2}, balancing experimental conditions). We computed the average probabilities to skip *p*_*skip,S*_ and to under-sample *p*_*us,S*_ from each group and run Kendall correlations tests between *p*_*skip*__,1_ and *p*_*us,*__2_, and *p*_*skip*__,2_ and *p*_*us,*__1_. This method was repeated 100 times and led to on average 67% (95%CI: [60.0,73.5]) of the tests being significant (*p* < .05) which is significantly higher than the false positive rate *α* = .05 (Binomial test, *p* < 2 × 10^–16^). This intricate balance between these strategies, both contributing to fluctuations, is further linked to the estimated level of risk aversion (β extracted from the best fitting model in each environment). Specifically, strategies favouring skipped trials are associated with lower β values (LMEM: *t* = –11.72, *p* < 2 × 10^–16^), while under-sampling is linked to higher β values (*t* = 5.93, *p* = 4.96 × 10^–9^) (Figure S8B). In the same way as above, we controlled for dependencies between estimated β values and probabilities of skipping and under-sampling by correlating these values extracted from different subsets of our data (β_1_, β_2_). For participants exhibiting significant fluctuations (at least 15% of the trials), results confirmed a negative correlation between β values and *p*_*skip*_ (proportions of significant tests: 100% [98.2,100], binomial test: *p* < 2 × 10^–16^) and a positive correlation between β values and *p*_*c < r*_) (proportions of significant tests: 94% [89.8,96.9], binomial test: *p* < 2 × 10^–16^). These findings are noteworthy for delineating the underlying factors guiding participants’ strategies, including individual differences related to risk tolerance.

## Allocation of resources within alternatives reflects an homogenous bias

### Humans tend to sample the alternatives homogeneously

**Figure 8:**
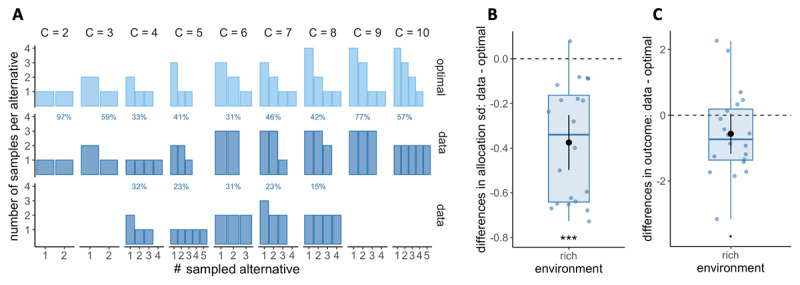
**Participants have a tendency to homogeneously allocate capacity amongst the sampled alternatives, but it has little impact on the outcome**. **A.** Number of samples allocated to each sampled alternative depending on the capacity allocated *C* in the rich environment. Upper panels: allocation of samples maximising the reward (optimal). Lower panels: most frequent allocations of samples observed across participants as a function of capacity. The allocations representing at least 50% of the trials are displayed and their likelihood is reported. **B.** Distribution of the differences between observed and optimal standard deviations of the distribution of samples among the selected alternatives in each environment (e.g. if C = 4 and 2 samples are allocated in a first alternative while the last 2 samples are each allocated in a second and third alternative, the standard deviation of this sample allocation would correspond to sd ({2, 1, 1}) ≈ 0.577). Note that more homogeneous distributions tend to lead to lower standard deviations. **C.** Distributions of the mean differences between observed and optimal outcomes in each environment. In the last two panels, dots represent participants and include all trials for which the optimal number of alternatives sampled doesn’t reflect a pure breadth strategy (*M*_*opt*_ < *C*– see Materials and methods for more details) and a capacity allocated up to 10. Below each distribution are presented results of one-sample Wilcoxon test (‘***’: *p* < 0.001 – B) and one-sample t-test (‘.’: *p* < 0.10 – C). N = 20, each data point averages 130 to 221 trials.

The optimal model also predicts the allocation of resources among each sampled alternative that maximises the reward. Particularly in the rich environment, where depth is favoured over breadth, predictions indicate that as capacity increases, it is optimal to allocate a different number of samples to each sampled alternative, thus avoiding ties (Figure 8A, upper panel). However, previous research has indicated an opposing tendency among participants, who preferentially tend to allocate their resources homogeneously among the sampled alternatives (Vidal et al., 2022). In this study, we replicated these findings and observed that participants favoured homogeneous allocation of resources in both the rich (Figure 8A, lower panels) and poor environments (Figure S9), over and above what would be expected by an optimal model. To characterise this bias towards homogeneous sampling, we focused on trials where a pure breadth strategy is not optimal (*M*_*opt*_ *< C*), indicating that homogeneous sampling is, therefore, suboptimal. Due to insufficient data to meet this condition in the poor environment, our analysis focused on the rich environment (see methods for more details). We computed the standard deviation of the ordered counts for each sample allocation, averaged for each participant, and compared it to the standard deviation of the optimal allocation. We observed that the standard deviation of sampled alternatives is significantly lower than that estimated from an optimal sample allocation (one-sample Wilcoxon test, *p* = 3.81 × 10^–6^, Figure 8B), indicating that participants have a bias towards distributing resources homogeneously among the selected alternatives.

### Sampling homogeneously has little impact on performance

Lastly, we investigated whether such deviation from optimality affected the participants’ reward (or outcome) in the task (Figure 8C). At each trial, the outcome is defined as the number of high-quality apricots among the 100 apricots purchased. We computed the differences between the observed and optimal outcomes (mean reward when following the ideal allocator) and observed a negative deviation from optimality (mean ± s.d.: –.56 ± 1.30), which was only marginally significant (*t*_19_ = –1.95, *p* = .066). The lack of influence on the outcomes might be attributed to the prevalent use of low capacity (2, 3, or 4 samples) for which, even in the rich environment, the optimal sample allocation does not substantially differ from a homogeneous allocation. In conclusion, participants exhibit a significant bias towards homogeneously sampling alternatives, as previously found (Vidal et al., 2022), compared to what is predicted by the optimal allocator model (Moreno-Bote et al., 2020). However, this deviation had a minimal impact on their performance.

## Discussion

Human behaviour is inherently variable, even when individuals engage in the same task over and over. For example, despite substantial practice and experience, professional basketball players will rarely reach free throw effectivity rates above 80%. Extensive research has explored the sources of this behavioural variability, often attributing it to stochastic neural processes (Faisal et al., 2008). Yet, recent evidence suggests that this variability should not be simply disregarded as noise (Garrett et al., 2013). Instead, it plays a significant role, notably in skill acquisition (Sternad, 2018) such as singing a new song (Ölveczky et al., 2005, 2011) or refining motor actions (Dhawale et al., 2017). Moreover, it fosters flexibility, facilitating exploration and the generation of novel behaviours, a particularly beneficial feature in uncertain environments (Kloosterman et al., 2019; Evans et al., 2019; Lee et al., 2023).

The present study delved into a less explored aspect of behaviour variability, focusing on the interplay between fluctuations in resource allocation and performance. We addressed whether behavioural variability during decision making is purely stochastic or else it is at least partially controlled, reflecting underlying strategies that go beyond the sole objective of maximising immediate reward. To investigate these questions, we employed a novel paradigm, the free Breadth-Depth dilemma (BD), which introduces a realistic scenario allowing for a better assessment of natural human decision-making. In this setting, individuals were tasked not only with determining which alternatives to explore in a block of consecutive choices but also the extent to which they explore them, granting them the freedom to strategise and plan fluctuations in resource allocation.

The BD dilemma involves active information sampling among multiple alternatives, in contrast to other paradigms that typically force binary choices (Daw et al., 2006; Weiss et al., 2021). Furthermore, BD dilemma diverges from the well-known exploration-exploitation dilemma (J. D. Cohen et al., 2007), where information seeking can only occur at the expense of forgoing the currently rewarding alternative. Notably, in the BD dilemma, the allocation of search capacity must be made without immediate feedback—a situation mirroring one of the core features of planning, where decision outcomes are often available after a delay, and changes of mind come at a cost, as in when modifying previous reservations of accommodation or transport.

### Summary of findings

Overall, this framework has proven to be relevant in uncovering and investigating fluctuations in capacity allocation. Indeed, when choice degrees of freedom are large in terms of variety of alternatives, and resource allocation can be freely distributed over alternatives and time, we observed significant deviations from an ideal allocator model. Most notably, participants distributed their initial capacity (*N_c_*) non-uniformly among the choices, allocating sometimes less than the capacity ratio *r* of the block and sometimes more, resulting in fluctuations (trials with *C* ≠ *r*, note that this definition includes skipped trials, where *C* = *0*). Crucially, the fluctuations observed in resource allocation cannot be simply attributed to passive stochastic processes. Indeed, these fluctuations were influenced by experimental manipulations, including the available capacity (*r*) and choice horizon (*N_trials_*), suggesting an active role and a rationality behind the observed fluctuations. Moreover, these fluctuations demonstrated a discernible structure, indicating a ‘save-for-later’ strategy (Figure 6) and adaptation to recent reward history (Figure 7). We further characterised these fluctuations in resource allocation by extending the ideal allocator model (Figure 8). Indeed, while human search behaviour has often been deemed suboptimal within the narrow framework of reward maximisation (Beck et al., 2012; Wyart & Koechlin, 2016), behavioural variability may serve functional roles beyond this context (Renart & Machens, 2014). We identified strategies that individuals follow which add up to the policy maximising immediate return: entropy seeking, risk avoidance and the pursuit of information-gain, and further indicate the controllability behind their behavioural variability. Additionally, individuals exhibit diverse resource allocation across choices, influenced both by the available capacity within the block and by choice horizon (Figure 5). Despite these deviations from optimal, participants managed to consistently maintain a well-balanced BD trade-off, reflecting an optimal equilibrium between the amount of resources allocated and the number of alternatives considered (Figure 4). In this sense, the fluctuations in resource allocation do not stymie the rationality of the observed behaviour, confirming again their non-passive origin. Below, we discuss the implications of these findings.

### Intentionality behind the fluctuations in resource allocation

The first crucial finding was that the fluctuations observed in resource allocation exhibit a discernible structure and should not be dismissed as mere noise. The results revealed that the occurrence of fluctuations varied depending on the experimental conditions. Participants were more inclined to skip sampling in a whole trial when search resources were scarce (low capacity), allowing them to garner larger amounts of information in other trials. The occurrence of skipped trials was also more pronounced when choice horizon was longer compared to shorter ones, suggesting a potential challenge in resource management (possibly reflecting Weber’s law). However, on average, participants retained some capacity until the beginning of the final trial in both short (mean ± sd: 9.86 ± .34 out of 10) and long blocks (19.51 ± .97 out of 20), indicating effective capacity management that enabled to sample information even in the last trial of the block. The higher rate of skipping in longer blocks, rather than indicating an earlier resource exhaustion, might signify a more extensive exploration of diverse strategies, capitalising on the additional time to apply acquired knowledge (Carstensen et al., 1999; Wilson et al., 2014).

Secondly, two sequential effects were found regarding how participants allocated their resources in choices, further underlying that the fluctuations observed respond to a controlled strategy. Participants seemed indeed to employ a ‘save-for-later’ strategy, allocating fewer resources (*C* < *r*) initially to conserve more resources for the subsequent choices (Figure 6). These findings underscore anticipation in participants’ resource allocation strategy. While this approach does not guarantee the optimal immediate reward, it may exhibit some advantages especially when applied to real-world scenarios. This strategy serves as a secure means of managing finite resources (such as a monthly budget or the time allocated for an exam), preventing early depletion, and averting detrimental outcomes, such as financial overdraw or exam failure. Moreover, it could result in having more flexibility to adapt to unexpected changes in the environment or to explore new strategies in the future.

In addition to being, at least partially, anticipated, evidence suggests that fluctuations in resource allocation also display flexibility by adjusting to recent reward history. Participants exhibit a greater likelihood of changing their strategy (shifting between sampling and skipping) following relatively lower outcomes (Figure 7A). What is more, the adaptive response to outcomes varies depending on the environmental context and seems to reflect violations in participants’ expectations.

### Individuals follow complex strategies beyond reward maximisation

At the level of single trials, we observe that individuals tend to allocate resources according to slightly simpler strategies than those predicted by the optimal model. For example, participants often distribute resources evenly across suppliers, a strategy that likely simplifies comparison across options when making a final choice (see Figure 8 and Vidal et al., 2022). While this within-trial approach provides insights into participants’ immediate resource allocation, it may miss the broader, more complex patterns that emerge over multiple trials. By examining resource allocation across trials, we can uncover these richer behavioural patterns, which reveal an underlying structure in participants’ fluctuations. This structure suggests that variability is, at least in part, a controlled process rather than mere noise. By extending the optimal allocator model we further identify three purposes behind those fluctuations which surpass immediate return maximisation and potentially offer valuable advantages in uncertain real-life scenarios.

### Entropy seeking

First, our findings revealed that participants actively seek to explore various strategies by maximising entropy. In our task, entropy is maximised by allocated many different capacities *C* in trials of a same block. The pursuit of entropy maximisation holds significance for the acquisition of novel information and the generation of innovative behaviours, intentionally observed in animals as a means of escaping predators (Evans et al., 2019) or contributing to cognitive flexibility (Dajani & Uddin, 2015; Uddin, 2021). This adaptive behavioural strategy plays a crucial role in adjusting to uncertain environments and fostering creative thinking and problem-solving. As mentioned in the previous section, adaptive responses to outcomes could be linked to violations in participants’ expectations. These two aspects, that is minimising surprises and maximising entropy, are pivotal ingredients of the free energy principle (Schwartenbeck et al., 2015). Such behavioural tendencies contribute to reducing prediction errors and constructing a more accurate model of the environment, facilitating rapid adaptation to potential changes. Moreover, the maximum occupancy principle, which favours entropy seeking, constitutes the core objective of newer theoretical frameworks modelling behaviour, offering an alternative perspective to reward maximisation (Ramírez-Ruiz et al., 2024).

### Risk avoidance

Secondly, our observations indicate that participants adopt strategies that may reflect individual attitudes towards risk. Indeed, under-sampling (allocating a capacity *C* inferior to the capacity ratio *r*) may have been employed to introduce fluctuations while mitigating the risk associated with skipping sampling and leaving some choices to chance (Figure S8). Research has demonstrated that individual differences in risk tolerance impact how limited resources are allocated, influencing performance (Tulloch et al., 2015). This bias affects the delicate balance between the short-term risk of loss in outcome and the long-lo benefits associated with learning. This underscores the necessity of incorporating individual features such as motivation (Zelick, 2007), curiosity traits (Risko et al., 2012), openness (Antinori et al., 2017) or beliefs associated with choice consequences (Loewenstein & Molnar, 2018) to enhance our understanding of the decision strategies humans adopt and why they deviate from a purely outcome-maximisation approach.

### Information benefit

Lastly, our findings also revealed that in rich environments where obtaining high outcomes is frequent, participants adopt strategies aimed at maximising the probability of identifying a single best-sampled alternative (no tie), thereby facilitating the final choice. Previous literature has demonstrated that humans value information independently from reward (Gottlieb, 2012; Bromberg-Martin & Monosov, 2020) and engage in informative sampling by exploring the more uncertain option (Wu et al., 2018; Gershman, 2019; Schulz et al., 2019; Wilson et al., 2021). In our study, participants follow specific strategies that contribute to reducing uncertainty about the final choice. Conversely, this pattern does not hold in the poor environment, where most rewards obtained are low. It is conceivable that, in this scenario, participants place a greater emphasis on maximising immediate outcomes. In contrast, in the rich environment, where high rewards are easily attainable, the task may appear less challenging, allowing participants to seek more useful information.

### Close-to-optimal BD trade-offs despite fluctuations

Although individuals fluctuated in the number of resources they allocated within each choice (trial), they were consistent in the number of alternatives they sampled for a given allocated capacity. Indeed, our observations indicated that the BD trade-off remained unaffected by both the horizon (block length, Figure 4B) and the number of available resources (capacity ratio, Figure 4C), suggesting a robust stability in the way breadth and depth are balanced. This underscores participants’ capacity to minimise variability, once again suggesting a level of control over behavioural fluctuations.

Additionally, we successfully replicated two important findings regarding the allocation of resources among multiple alternatives (Vidal et al., 2022). First, our study revealed that participants follow a heuristic power-law sampling strategy, aligning with the optimal BD trade-offs (Moreno-Bote et al., 2020) and adapting effectively to the environmental richness (Figure 4A). Crucially, we did not observe any significant disparities between the optimal BD balance and participants’ behaviour in both environments, as opposed to prior findings (Vidal et al., 2022). This difference might be attributed to the relatively lower capacity ranges employed in our study, which is conceivably easier to manipulate accurately. However, our paradigm also diverges by granting participants greater control over their information sampling, enabling them to choose not only what will be explored but also to what extent. Encouraging active engagement in a task, through methods like active learning, has been demonstrated to enhance performance significantly (Voss et al., 2011; Freeman et al., 2014) and may have played a role here.

### Homogenous allocation within alternatives

Our study also replicated previous findings indicating that participants tend to distribute their capacity uniformly among the sampled alternatives (Figures 8 and S9). The origin of this bias is challenging to ascertain. It may arise from the increased ease of comparing fractions with a common denominator, thereby reducing cognitive load. Homogenous sampling could also emerge as a means to ensure that alternatives are equally risky, as they carry the same amount of information, aligning with humans’ preference to standardise the uncertainty associated with the alternatives (Schulz et al., 2019; Wilson et al., 2021; Alméras et al., 2021). Finally, it may also stem from the visual presentation of the design and symmetry being aesthetically pleasing for humans (Attneave, 1955). Further investigations are necessary to disentangle these potential causes, but the study already confirms the robustness of this effect.

### Exploring the relationship between fluctuations and performance

Although our results demonstrate that the observed behavioural fluctuations exert only a modest effect on immediate task performance (see also the *Limitations* section below), the underlying complex strategies may nonetheless serve alternative latent objectives that are not captured by metrics of immediate reward, such as facilitating long-term learning or preserving cognitive flexibility enabling, for example, better responsiveness to changes in the environment. In addition, fluctuations resulting from future intentions or past rewards may contribute to reinforcing participants’ sense of agency via the exertion of greater cognitive effort (Bussche et al., 2020), thereby influencing their task engagement and subsequent performance (van der Wel et al., 2012; Hon & Yeo, 2021). Notably, participants exhibiting increased fluctuations appear to adhere more closely to the optimal BD trade-off, particularly in the rich environment where an optimal balance may be more difficult to grasp as diverging from pure breadth (Figure S10). Although, our data is limited to properly assess the presence of this effect, this study could pave the way for investigating the impact of endogenous versus induced variability in resource allocation on participants’ task engagement and on the adoption of strategies closer to optimality. Indeed, many studies exploring human information processing employ paradigms where participants lack control over information sampling (such as dot motion tasks: Glaze et al., 2015; sequences of stimuli: De Lange et al., 2010; Wyart et al., 2012; Cheadle et al., 2014), which does not reflect the decision-making reality outside the laboratory. In real-world scenarios, individuals actively choose where to look at, what to listen to, or what to click on. Information acquired through one’s actions, in contrast to passively received, has been shown to enhance performance (Ariely, 2000; Voss et al., 2011; Freeman et al., 2014), particularly through active hypothesis testing (Markant & Gureckis, 2014; Markant et al., 2016).

### Limitations and further research

In summary, our findings highlight the utilisation of controlled strategies that, while diverging from optimality, exhibit features of anticipation and adaptation. These strategies require more cognitive effort than, for example, the homogeneous allocation of resources across choices. Actually, although effort is generally aversive (Kool et al., 2010; Kurzban, 2016), humans willingly engage in activities that demand increased effort, such as participating in charity runs, solving challenging sudoku puzzles, or assembling their own furniture (Inzlicht et al., 2018). Effort, therefore, is not solely balanced against the benefit associated with an action or computation (Kurzban et al., 2013) but can have intrinsic positive value and add to the perceived value of outcomes. Studies have shown that individuals are inclined to undertake more effortful actions when experiencing boredom. While we did not monitor participants’ motivational or attentional states in our study, their interest in the task and their perception of its difficulty may have influenced their likelihood to pursue more complex explorative strategies (Milyavskaya et al., 2021; Wu et al., 2023). For example, boredom has been identified as a factor promoting information-seeking behaviour (Geana & Daw, 2016; Danckert, 2019; Agrawal et al., 2022). Additionally, we did not observe a significantly higher number of skipped trials in the poor environment compared to the rich one, contrary to predictions from the optimal model. Indeed, although the proportions in which fluctuations exhibited by participants deviated from optimality significantly impacted their averaged reward, the loss in outcome was relatively small (Figure S4) and might not have been sufficient to incentivise a notable modulation in participants’ sampling strategy. Manipulating the magnitude of rewards may be interesting to emphasise the gap between optimal and sub-optimal strategies (opportunity cost) and investigate whether humans are able to exert more effort to implement better sampling strategies and adapt their level of fluctuations.

Our study has other limitations associated with the assumptions of the ideal allocator model. Firstly, the model assumes that participants have complete knowledge of the posterior distribution of the success probability of the alternatives, which, in reality, they do not possess. Nevertheless, our findings indicate that participants can accurately infer the environment richness, as evidenced by their BD trade-offs aligning with optimality. Furthermore, we interpret deviations from optimality in resource allocation across trials not as misjudgments of the environmental context, but as evidence of participants pursuing goals beyond simple reward maximisation. Secondly, in terms of resource allocation across choices, our extended model performs well in predicting participant behaviour. However, the information benefit is estimated based on the optimal allocation of resources within a choice (how many alternatives are sampled – BD trade-off) and within each alternative (how many samples are in each alternative), and not on the observed allocations. While this assumption holds predominantly true in the poor environment, it is less accurate in the rich environment, especially for larger capacity allocations (*C* > 4). Notably, such trials are infrequent (8%), bolstering our confidence that this assumption does not undermine the robustness of our results.

Moreover, while our paradigm enables control over search capacity by manipulating the number of samples available, it does not incorporate the cost of sampling, a factor known to influence human and non-human primate sampling strategies (Petitet et al., 2021; Drugowitsch et al., 2012). In our experiment, all samples are standardised with the same cost (one coin = one sample), yet they may vary in computational costs, considering that allocating a sample to maximise potential information depends on the number and manner in which previous samples have been allocated. While the cost-benefit structure may have had a limited impact on our results due to the predominant allocation of low capacity, exploring the effect of fluctuations on performance (e.g., the BD trade-off) would necessitate accounting for the cost of sampling to comprehensively understand the goals and constraints underlying human search strategies.

### Conclusions

To conclude, the results illustrate that human resource allocation behaviour is characterised by more variability than what the optimal model – maximising only immediate return – would anticipate, whilst still rendering a consistent near-optimal BD strategy. These findings shed light on the nature of behavioural fluctuations that help balancing the level of risk at stakes by enhancing information gathering, and the generation of diverse strategies whilst maintaining near-optimal performance. This may ultimately reflect advantageous and contextually relevant behaviours which imply anticipation, such as saving resources for later important choices, and flexible adaption based on past rewards and environmental conditions. Essentially, this work presents a novel framework that has proven useful to gain insights into the origins of behavioural fluctuations in the allocation of resources, revealing a structure that goes beyond a purely stochastic process.

## Data Accessibility Statement

Data is accessible at this link: https://osf.io/dhxjb/overview.

## Additional File

The additional file for this article can be found as follows:

10.5334/joc.467.s1 Supplementary Figures. Figures S1–S10.
