# Supplementary material for: Fluctuations in Sequential Many-Alternative Decisions Reveal Strategies Beyond Immediate Reward Maximisation
Source: J Cogn. 2025 Nov 18;8(1):55. doi: 10.5334/joc.467 (PMC12636281; doi:10.5334/joc.467)
Supplement: Supplementary Figures. — Figures S1–S10. [file joc-8-1-467-s1.pdf]

Supplementary Figures

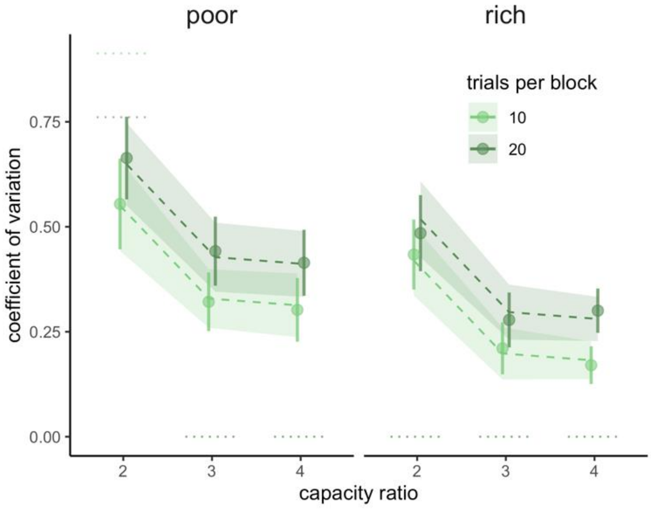

Figure S1. **Participants capacity allocation fluctuates more when little capacity is available and with larger horizons.** Coefficient of variation (CV) of the capacity allocated over the block depending on the capacity ratio *r*, block length (colours) and the environment (poor or rich). Vertical bars represent s.e.m. of the data, while dashed lines and shaded areas represent respectively the predicted averages and s.e.m. using LMEM. Dotted horizontal segments represent the CVs predicted by the optimal model. LMEM results reveal a significant effect of the ratio ($\chi_1^2 = 114.93, p < 2.2 \times 10^{-16}$) and block length ($\chi_1^2 = 23.71, p = 1.12 \times 10^{-6}$). No significant effects of the environment ($\chi_1^2 = 1.99, p = .16$), nor an interaction between the block length and the capacity ratio were found ($\chi_1^2 = .71, p = .70$). [back to Methods]

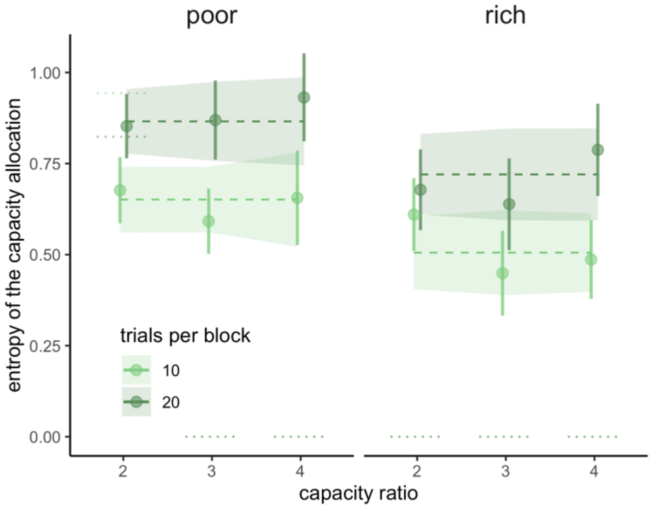

Figure S2. **The entropy of participants capacity allocations does not significantly fluctuate depending on the experimental conditions.** Entropy of the capacity allocated over the block depending on the capacity ratio *r*, block length (colours) and the environment (poor or rich). Vertical bars represent s.e.m. of the data, while dashed lines and shaded areas represent respectively the predicted averages and s.e.m. using LMEM. Dotted horizontal segments represent the CVs predicted by the optimal model. LMEM results reveal a significant effect of the block length only ($\chi_1^2 = 43.91, p = 3.44 \times 10^{-11}$). No significant effects of the environment ($\chi_1^2 = 1.35, p = .25$), the capacity ratio ($\chi_2^2 = 4.56, p = .10$), nor an interaction between the block length and the capacity ratio were found ($\chi_1^2 = 4.57, p = .10$). [back to Methods]

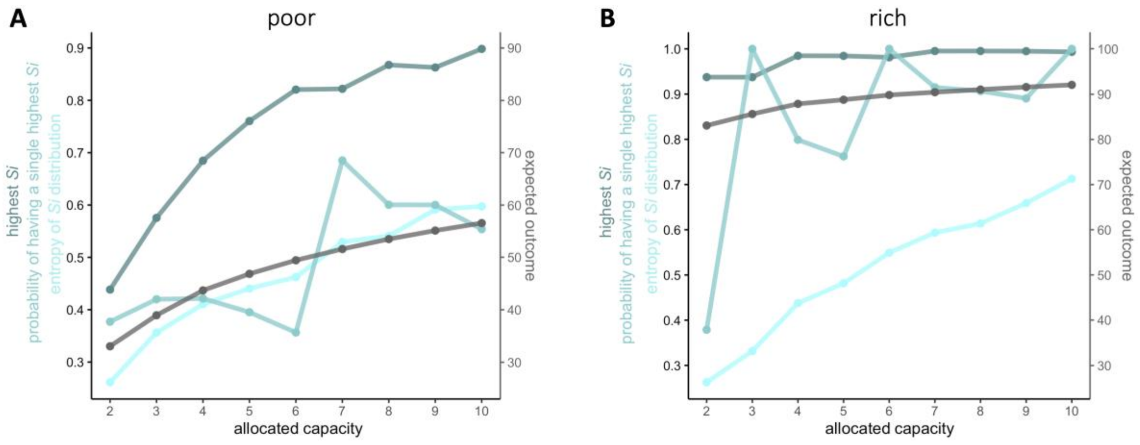

Figure S3. The information benefits evolve with capacity differently from the expected outcome. A-B. Mean highest sampled probability (S_i) of the sampled alternatives (I_{max}, darker sea green), mean probability that this highest sampled probability S_i correspond to a single sampled alternative and not several ones (I_{single}, medium sea green) and mean entropy of S_i distribution ($I_{entropy}$, lighter sea green). The mean expected outcome when selecting the sampled alternative with the highest normative outcome V_i is plotted in dark grey. The poor (**A**) and rich (**B**) environments are presented respectively in the left and right panels. [back to Methods]

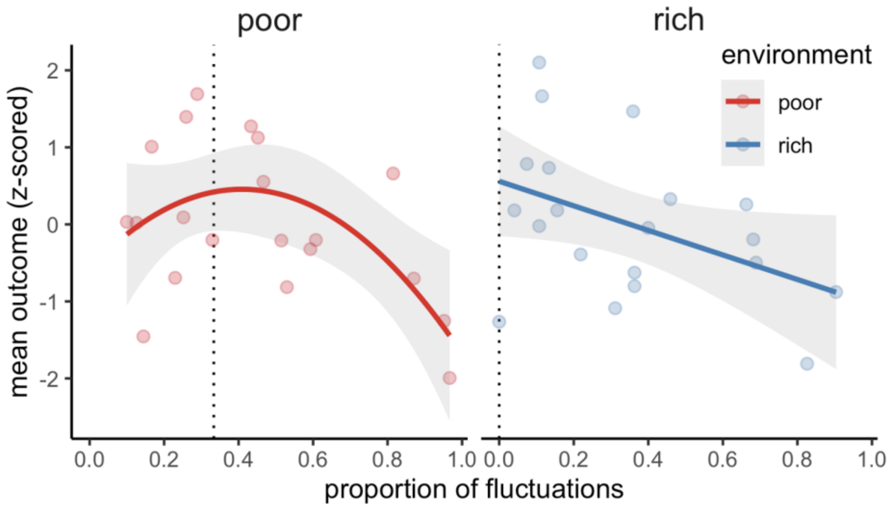

Figure S4. Participants outcomes are affected by their level of fluctuations in the resources allocation similarly as predicted by the optimal model. Participants mean outcomes received (z-scored by environment) depending on individuals' level of fluctuations, measured by the proportions of trials with the allocated capacity C different from the capacity ratio r. The vertical dotted lines correspond to the optimal proportions of fluctuations in each environment. The coloured lines represent the model best fitting the data (second-order polynomial or linear). Each data point corresponds to the averaged of outcomes and fluctuations over all the trials, for each participant. [back to Results] [back to Discussion]

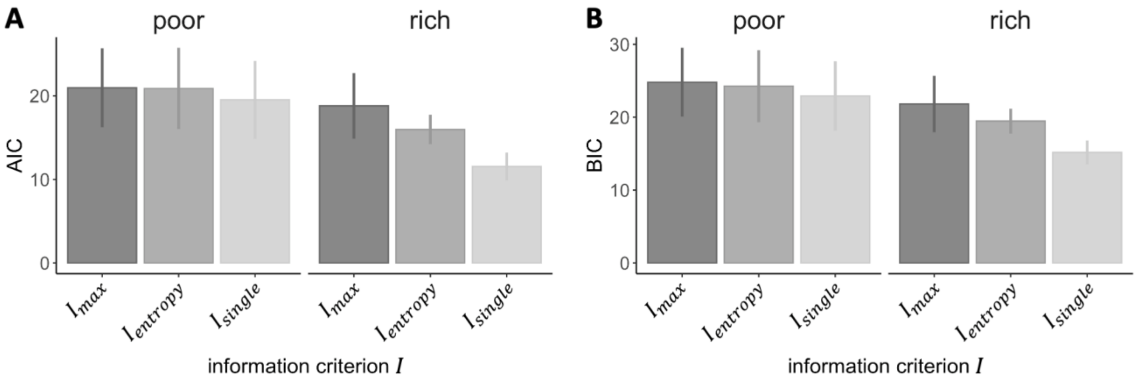

Figure S5. **The information criterion maximising the probability to obtain a single best sampled alternative is predicting the participants search strategy the best.** Averaged AIC (**A**) and BIC (**B**) across participants in the poor and rich environments estimated by fitting the data within each block using the full model (α, β and γ different from zero) with the. Three different information criterions (see Methods). [back to Results]

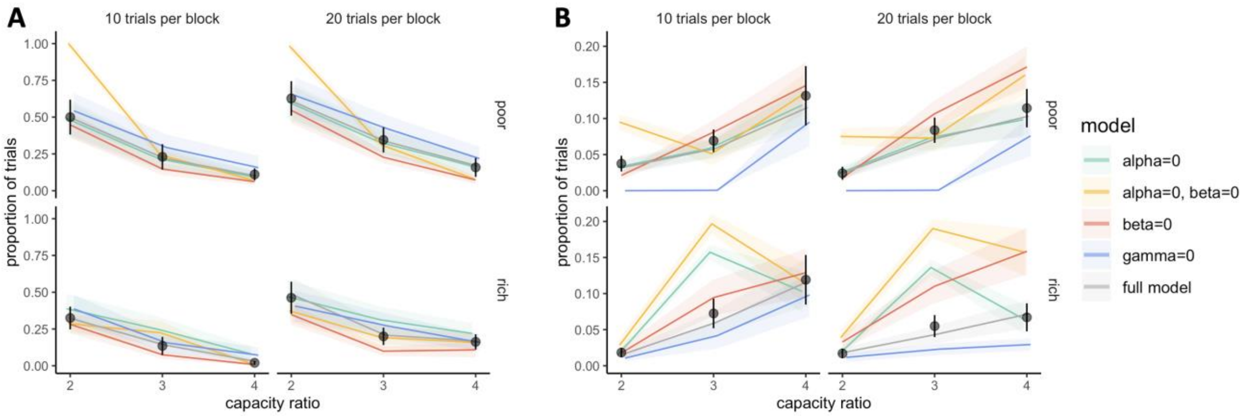

Figure S6. Fluctuations in resource allocations among choices are well predicted by the extended optimal models (see Eq.1). Proportion of skipped trials (C=0, **A**) and trials with a non-null capacity allocated inferior to the ratio ($0<C<r$, **B**) depending on the capacity ratio, block length and environment richness. Black points represent the averaged observed probabilities across participants and vertical bars the s.e.m. Colours lines represent the averaged fitted probabilities for each model and the shaded areas the s.e.m. across participants. [back to Results]

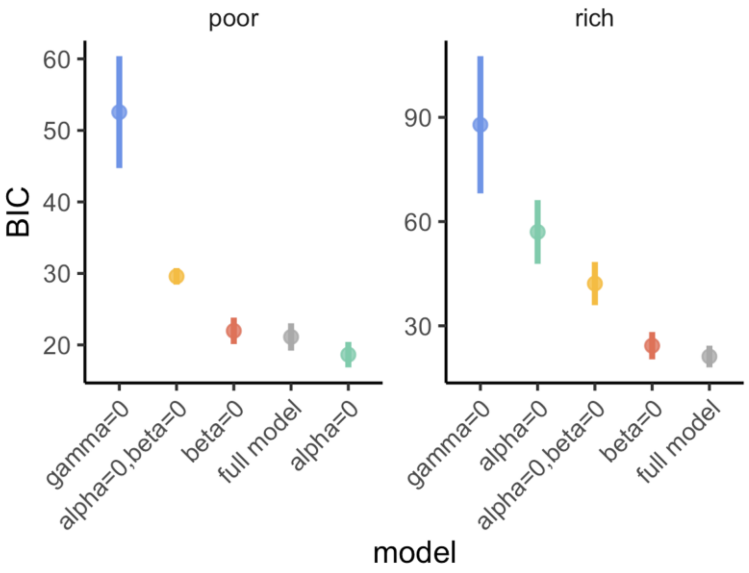

Figure S7. Averaged BIC across participants in the poor (left) and rich (right) environments estimated by fitting the data within each block using the five different models. Vertical bars represent s.e.m. [back to Results] [back to Methods]

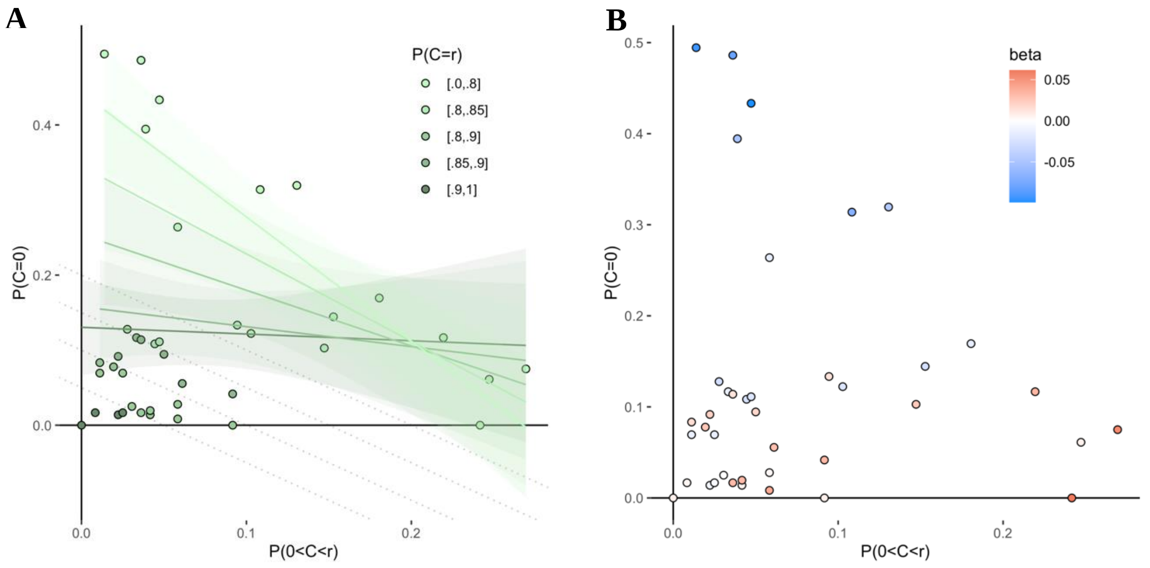

Figure S8. Participants whose resource allocations fluctuate follow a strategy which balances skipping sampling (C=0) and allocating little capacity (0<C<r) depending on individual fitted risk aversion (β). A. Averaged individual probabilities to skip sampling depending on the probability to allocate a capacity inferior to the capacity ratio *r*. The colours represent the averaged individual probabilities to allocate a capacity equal to *r* (not fluctuating) and are also separated by the diagonal grey dotted lines. The coloured lines represent the correlation between both probabilities (skipping and allocating a capacity inferior to the ratio) including all participants (darker green) or participants with a minimum fluctuating probability (lighter greens). Shaded areas represent 95% CI. Results of these correlations are as follow: all participants: $tau = .12, p = .28$, N=40; participants with P(C=r) < .95: $tau = -.01, p = .92$, N=36; P(C=r) < .90: $tau = -.24, p = .097$, N=25; P(C=r) < .85: $tau = -.46, p = .006$, N=19; P(C=r) < .80: $\rho = -.86, p < 2 \times 10^{-16}$, N=16). **B.** Idem as panel A but dots are coloured depending on averaged individual estimated betas parameters of the best fitting model (full model in the rich environment and model with alpha=0 in the poor). Fitted beta parameters are found to decrease with the proportion of skipped trials (LMEM: $t = -11.72, p < 2 \times 10^{-16}$) and increase with the proportion of trials with an allocated capacity inferior to the ratio (0<*C*<*r*) ($t = 5.93, p = 4.96 \times 10^{-9}$), revealing that participants with a large proportion of trials with 0<C<r are also characterised by a higher risk aversion (high betas). [back to Results] [back to Discussion]

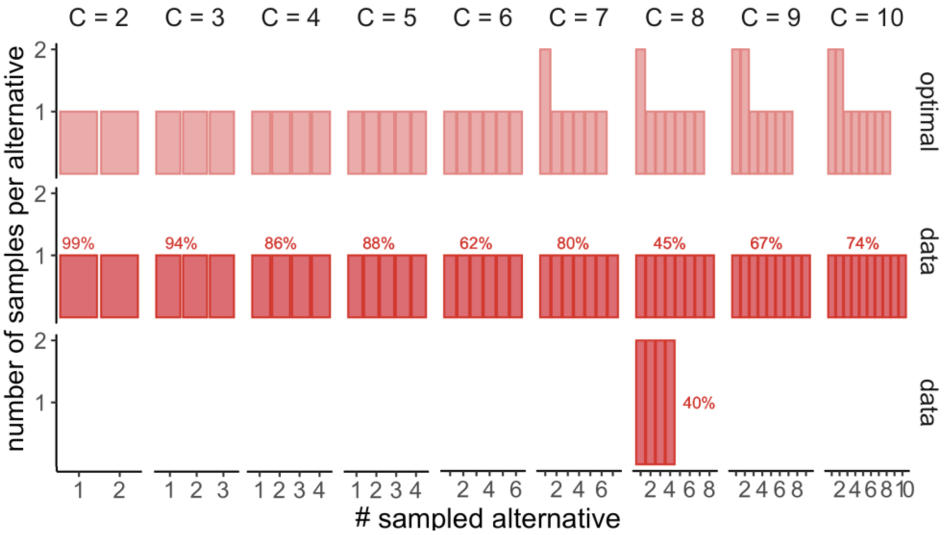

Figure S9. Participants have the tendency to homogenously allocate capacity amongst the sampled alternatives. Number of samples allocated to each sampled alternative depending on the capacity allocated *C* in the poor environment. Upper panels: allocation of samples maximising the reward (optimal). Lower panels: most frequent allocations of samples observed across participants as a function of capacity. The allocations representing at least 50% of the trials are displayed and their likelihood is reported. [back to Results] [back to Discussion] [back to Methods]

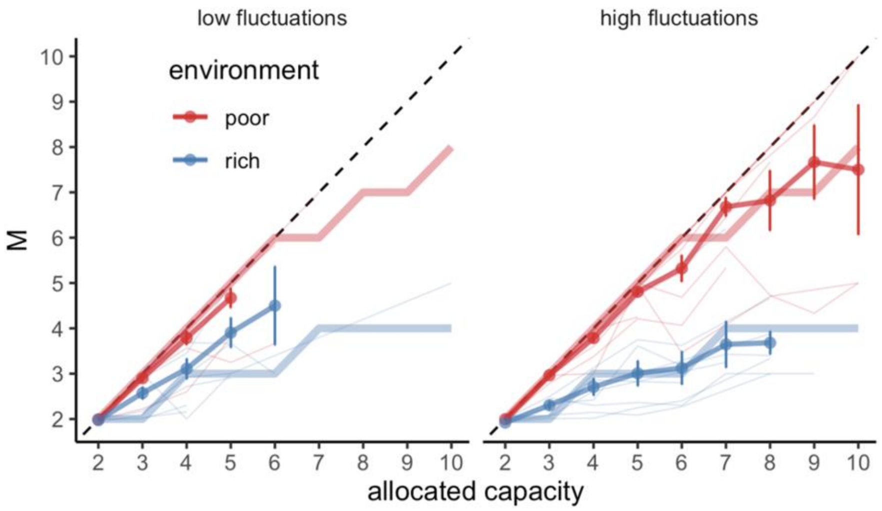

Figure S10. Fluctuating in the resource allocation seem to be associated with close-to-optimal BD trade-offs. Number of alternatives sampled (M) depending on the capacity allocated, the environment richness (colours) and the proportion of fluctuations (median split on individuals' proportions of trials with C ≠ r). Group average and s.e.m. are plotted above individual data (thin light lines) and optimal values of M (thick light lines). Dashed lines indicate unit slope line. N=20 per environment. [back to Discussion]
